# Ginsenosides in hepatocellular carcinoma: preclinical evidence, mechanisms, and clinical implications

**DOI:** 10.3389/fphar.2026.1973780

**Published:** 2026-10-22

**Authors:** Caixin Yu, Xinyue Zhu, Shiqi Liu, Yun Ding, Hong Fu, Yan Li, Meichen Gai, Mengyao Tan, Zhongwei Zhao

**Affiliations:** 1 College of Special Education, Changchun University, Changchun, China; 2 School of Medicine, Changchun University of Science and Technology, Changchun, China; 3 Department of Internal Medicine, Guang’anmen Hospital, Chinese Academy of Chinese Medical Sciences, Beijing, China; 4 Qingdao Hospital of Traditional Chinese Medicine, Qingdao, China

**Keywords:** apoptosis, autophagy, ginsenosides, hepatocellular carcinoma, nanomedicine, therapy, tumor microenvironment

## Abstract

Hepatocellular carcinoma (HCC) remains a major cause of cancer mortality worldwide, with limited effectiveness of current strategies and persistent challenges in early detection and outcome prediction. This review summarizes the preclinical evidence supporting the therapeutic potential of ginsenosides from Panax ginseng—particularly Rh2 and Rg3—in HCC cellular and animal models. Mechanistically, the antitumor effects are discussed in relation to induction of apoptosis, modulation of autophagy and other cell-death–associated signaling, and reprogramming of the tumor microenvironment, alongside evidence for suppression of metastatic behavior and enhancement of antitumor immunity. Finally, the review provides clinical implications by highlighting how these mechanistic findings may inform future translation, including opportunities for rational combination regimens and drug delivery approaches to overcome key translational bottlenecks—particularly low bioavailability and the lack of reliable biomarkers for response prediction and patient stratification—to optimize clinical application. Overall, the available preclinical data suggest that ginsenosides are promising candidates for improving HCC management, while emphasizing the need for further mechanistically rigorous and translational studies to support clinical optimization.

## 1 Introduction

Hepatocellular carcinoma (HCC) is the most common primary liver malignancy, arising from hepatocytes on a background of chronic liver injury. Clinically, HCC is classified by etiology (e.g., hepatitis B virus–related, hepatitis C virus–related, alcohol-related, and metabolic dysfunction–associated steatotic liver disease–related), pathological growth and differentiation patterns (e.g., trabecular, solid, pseudoglandular, and scirrhous), and stage/management, which are commonly summarized by staging systems such as Tumor–Node–Metastasis (TNM) and Barcelona Clinic Liver Cancer (BCLC) (integrating tumor burden, liver function, and performance status) (Alawyia and Constantinou, 2023). Epidemiologically, the global burden of HCC continues to rise, driven by the long-term progression of chronic liver diseases, changes in metabolic risk factors, and differences in surveillance and early diagnosis. This translates into substantial public health and healthcare economic pressure due to the need for repeated monitoring, complex multimodal treatments, and frequent recurrence management (Toh et al., 2023; Frager and Schwartz, 2020). Although therapeutic strategies have evolved—combining curative modalities (surgery, transplantation, local ablation/radiation for selected patients) with locoregional and systemic therapies (transarterial chemoembolization (TACE)/transarterial radioembolization (TARE), targeted agents, and immunotherapy) alongside antiviral therapy and risk-factor management—several gaps remain. Key limitations include insufficient sensitivity and specificity of available biomarkers for early detection and prognostication, inadequate tools to predict treatment response and recurrence, and persistent challenges with resistance and disease heterogeneity. Therefore, further mechanistic and translational research is needed to improve prevention, optimize therapy, and ultimately enhance patient outcomes (Chan et al., 2024; European Association for the Study of the Liver, 2025; Nagalapuram et al., 2025).

Ginsenosides are naturally occurring triterpenoid saponins that represent the major bioactive constituents of Panax ginseng, Panax quinquefolius, and Panax notoginseng. Structurally, ginsenosides are commonly classified as protopanaxadiol (PPD)-type compounds, including Rb1, Rb2, Rc, Rd, Rg3, Rh2, and Rk1, and protopanaxatriol (PPT)-type compounds, including Rg1, Rg2, and Re. In addition, some ginsenoside-related compounds are metabolites or processing-derived derivatives, such as compound K (CK) (Z et al., 2021; Chopra et al., 2023; Paik et al., 2023). In this review, the term “ginsenosides” refers to the specific Panax-derived chemical entities and administered preparations reported in each included study, including parent ginsenosides, metabolites or processing-derived derivatives, total-ginsenoside preparations/extracts, mixtures, and ginsenoside-based delivery systems. Biological comparability is therefore interpreted within the stated chemical boundaries of identity, stereochemistry, purity, and formulation rather than assuming equivalence across chemically distinct entities. Evidence was primarily synthesized from HCC-relevant models; studies using hepatoblastoma-derived cells or broadly labeled liver-cancer models were categorized separately and interpreted with limited transferability to established HCC settings. Pharmacologically, ginsenosides exhibit pleiotropic effects including anti-inflammatory activity, antioxidant effects, modulation of apoptosis and autophagy, immune regulation, and influences on metabolic reprogramming and key signaling networks (Paik et al., 2023; Li et al., 2023). Their therapeutic potential has also been reported across multiple organ systems and diseases, including conditions characterized by chronic inflammation and metabolic dysregulation, as well as selected cardiovascular and neurodegenerative disorders (Z et al., 2021; Yang et al., 2023a). Importantly, accumulating evidence in recent years suggests that ginsenosides may confer potential anti-HCC benefits by suppressing tumor growth and survival, regulating autophagy and apoptosis, and reshaping the tumor microenvironment (Li et al., 2023; Yao et al., 2026). These findings offer a new perspective for understanding HCC development and for refining therapeutic concepts. Accordingly, this review summarizes current preclinical evidence for ginsenoside-based anti-HCC effects, covering both cellular and animal studies, and further consolidates the proposed mechanisms underlying these observations. Finally, from a clinical standpoint, this review presents our treatment insights and research perspectives to propose novel ideas for improving the prevention and management of HCC.

## 2 Literature search and study selection

PubMed and Web of Science were searched from database inception to August 2026 using the terms “ginsenoside” and “hepatocellular carcinoma,” with additional searches for Rh2, Rg3, compound K, Rk1, and other relevant ginsenosides combined with HCC-related terms such as “liver cancer.” Mechanism- and phenotype-related keywords, including apoptosis, autophagy, ferroptosis, migration/invasion, epithelial–mesenchymal transition (EMT), tumor microenvironment, and immune modulation, were added where appropriate. A total of 495 records were identified. After duplicate removal, 338 records were screened by title and abstract. Following full-text assessment, 111 full-text articles were assessed for eligibility, and 86 studies were included in the qualitative synthesis. A total of 495 records were identified. After duplicate removal, 338 records were screened by title and abstract. Following full-text assessment, 111 full-text articles were assessed for eligibility. We did not conduct a formal risk-of-bias or study-quality assessment because the included preclinical and clinical studies were highly heterogeneous in experimental designs (e.g., different models, dosing regimens, and outcome reporting), and this review aimed to qualitatively synthesize mechanistic and translational findings rather than to quantitatively pool effect sizes across studies. Ultimately, 86 studies were included in the qualitative synthesis. Only English-language publications were included. Conference abstracts were excluded because they often provide limited methodological and experimental details required for eligibility assessment. (Supplementary Figure S1).

Studies were eligible if they evaluated ginsenosides in HCC-relevant cellular and/or animal models and/or human clinical studies, reporting (i) antitumor effects in humans and/or (ii) antitumor effects and related mechanistic findings in preclinical models. Non-HCC studies, duplicate reports, reviews/editorials, conference abstracts with insufficient experimental information, and studies lacking relevant experimental data were excluded. For each included study, we extracted key information regarding ginsenoside identity and preparation, experimental model, treatment conditions, major antitumor outcomes, and the proposed mechanisms, and we summarized the main findings in Supplementary Tables S1–S5. Given substantial heterogeneity across study designs, models, treatment regimens, and outcome measures, the evidence was synthesized qualitatively.

To ensure comparability and to avoid pooling biologically distinct entities, we defined explicit chemical boundaries and interpreted evidence accordingly. Chemically defined parent ginsenosides were treated as distinct from metabolites or processing-derived derivatives. Total-ginsenoside preparations or extracts, as well as mixtures, were analyzed as the tested preparation rather than as equivalent to any single corresponding parent compound, because composition, potency, and bioavailability may differ. Ginsenoside-based delivery systems (e.g., nanoformulations) and combination regimens were also considered within the boundary of the administered formulation or regimen, and findings were not regarded as interchangeable with those of the corresponding free parent compound. This approach was used consistently across the review to prevent an overgeneralized inference that all “ginsenosides” share identical activity and translational implications. We also applied disease-model boundaries to restrict how evidence from different liver-cancer settings was interpreted. The primary focus was evidence derived from HCC-relevant models. Studies using hepatoblastoma-derived or broadly labeled liver-cancer models (e.g., HepG2) were retained only as supportive, liver-tumor–relevant evidence with limited transferability to established HCC settings, and they were not treated as components of the main HCC evidence base. Accordingly, these studies were flagged and interpreted cautiously, categorized separately, and their findings were not considered fully equivalent to results from established HCC models. Across all model categories, mechanistic interpretations were restricted to what was directly reported within the specific model context, thereby improving the traceability of conclusions and reducing the risk of misattribution across heterogeneous preclinical evidence.

## 3 Potential molecular mechanisms underlying the anti-HCC effects of ginsenosides

### 3.1 Immune and inflammatory modulation by ginsenosides

Inflammatory signaling and immune suppression are closely interconnected in the HCC microenvironment. Available preclinical studies suggest that different ginsenosides may converge on the attenuation of tumor-promoting inflammation and the partial restoration of antitumor immune activity, although their reported proximal targets and experimental contexts differ. The current evidence is derived predominantly from cellular and animal models; therefore, the proposed immunomodulatory effects should be interpreted as preclinical mechanistic observations rather than established clinical benefits. At the level of inflammation-associated signaling, several ginsenosides have been linked to modulation of the gut–liver axis and major inflammatory pathways. Rk3, a processing-derived ginsenoside, was reported to attenuate liver tumor development in a diethylnitrosamine (DEN)/carbon tetrachloride (CCl4)-induced mouse model, accompanied by amelioration of gut microbiota dysbiosis, reduced inflammatory cytokine expression, and inhibition of lipopolysaccharide (LPS)–Toll-like receptor 4 (TLR4)-associated signaling (Qu et al., 2021). In an LPS-induced inflammatory context, Rh4 was reported to suppress histone deacetylase 4 (HDAC4)/interleukin-6 (IL-6)/signal transducer and activator of transcription 3 (STAT3) signaling and to reduce the expression of glycolysis-related molecules, including lactate dehydrogenase A (LDHA) and glucose transporter 1 (GLUT1), concomitant with decreased proliferation and migration-associated phenotypes (Jiang et al., 2023). These studies suggest that certain ginsenosides may influence inflammation-associated liver tumor progression through distinct upstream mechanisms.

Other compounds have been associated with nuclear factor-kappa B (NF-κB)- or cyclooxygenase-2/prostaglandin E2 (COX-2/PGE2)-related signaling. Compound K (CK), a ginsenoside metabolite, was reported to attenuate nuclear factor-kappa B (NF-κB)-associated signaling and matrix metalloproteinase 2/9 (MMP2/9)-related invasive phenotypes. In particular, CK was proposed to target Annexin A2 and interfere with its interaction with nuclear factor-kappa B p50 (NF-κB p50), thereby reducing downstream NF-κB signaling and promoting apoptosis-related responses (Ming et al., 2011; Wang et al., 2019). Rh2 and its structurally modified octyl ester derivative, Rh2-O, were reported to inhibit migration- and invasion-associated phenotypes through the c-Jun/cyclooxygenase-2/prostaglandin E2 (c-Jun/COX-2/PGE2) axis; the partial reversal of these effects by exogenous PGE2 or c-Jun overexpression supports the involvement of this pathway (Hu et al., 2023). However, the activity of Rh2-O should be interpreted as evidence for the tested derivative rather than as equivalent to the activity of free Rh2.

In addition to suppressing inflammatory signaling, several studies indicate that ginsenosides may influence immune-cell function within the tumor microenvironment. Rg3 was reported to modulate Kupffer-cell-associated immune phenotypes and to reduce intratumoral accumulation of myeloid-derived suppressor cells (MDSCs) in experimental HCC models (Park et al., 2026; Zhang et al., 2024). CK was also reported to downregulate enhancer of zeste homolog 2 (EZH2) and promote dendritic cell–NK cell-associated antitumor activity, thereby alleviating immunosuppressive features of the tumor microenvironment in the tested model (Ye et al., 2025). These findings suggest that the immune effects of ginsenosides may extend beyond direct actions on tumor cells; nevertheless, direct comparisons across compounds and models remain limited.

Rh2 and Rh1 provide further evidence for the modulation of adaptive antitumor immunity. In a Hepa1-6/splenocyte co-culture system, Rh2 increased CD8^+^ T-cell effector-associated markers, including cluster of differentiation 107a (CD107a) and interferon-gamma (IFN-γ), while reducing programmed death-ligand 1/programmed death-1 (PD-L1/PD-1) signaling, immunosuppressive cytokines (transforming growth factor-beta 1 (TGF-β1), interleukin-6 (IL-6), and interleukin-10 (IL-10)), and regulatory T-cell proportions (Park et al., 2026). In mouse and *ex vivo* HCC models, Rh1 was reported to modulate glucocorticoid receptor (GR)-associated signaling, increase MHC-I expression, promote dendritic-cell maturation, and enhance CD8^+^ T-cell activation. Rh1 also enhanced the antitumor activity of lenvatinib in the tested model (Wang et al., 2024).

Overall, the available evidence indicates that ginsenosides may act at multiple points along the inflammation–immunosuppression–immune-effector axis. Rk3 and Rh4 have mainly been linked to inflammation-associated gut–liver and IL-6/STAT3 signaling, whereas CK, Rh2, and Rh2-O have been associated with NF-κB- or COX-2/PGE2-related pathways. Rg3, Rh2, Rh1, and CK have additionally been reported to affect immune-cell subsets or immune-checkpoint-associated signaling. Despite this convergence, the chemical forms, models, and mechanistic endpoints differ substantially among studies; thus, these findings should not be interpreted as demonstrating equivalent immunomodulatory activity among all ginsenosides.

### 3.2 Apoptosis pathways involved in ginsenoside-mediated anti-HCC effects

Following ginsenoside exposure, liver tumor cells are often reported to involve apoptosis as a terminal outcome. Mechanistically, reported studies are consistent with a stress-integrated framework in which ginsenosides are reported to be associated with (i) mitochondria-mediated (intrinsic) apoptosis, (ii) death-receptor–linked (extrinsic) apoptosis, and (iii) ER-stress/UPR signaling associated with caspase activation amplification-like changes.

A recurring mechanism is the mitochondrial intrinsic pathway, described as being associated with redox imbalance and mitochondrial destabilization. In Hep3B cells, KRG (Korean red ginseng) and its major constituents ginsenoside Rg3 (Rg3) and ginsenoside Rh2 (Rh2) increase intracellular reactive oxygen species (ROS) while reducing mitochondrial membrane potential, accompanied by cytochrome c release, BCL2-associated X protein (Bax) induction, and caspase-3 activation, which is consistent with a mitochondria-centered apoptotic program in this model (Park et al., 2012). Rh2 has been reported to further support this model by increasing Bcl-2-associated X protein (Bax)/Bcl-2-antagonist/killer (Bak) translocation, with cytochrome c release and downstream caspase activation observed in hepatoma cells (Guo et al., 2012). Notably, Rh2 has been reported to activate caspase-dependent death even when Bcl-2 is overexpressed, suggesting that the apoptotic execution machinery may still function under enforced anti-apoptotic buffering (Park et al., 1997). Similar mitochondria-coupled apoptosis has been described for protopanaxadiol-related metabolites and for CK, where mitochondrial depolarization and caspase activation occur alongside DNA damage and apoptotic progression (Oh and Lee, 2004; Zheng et al., 2014).

In addition to intrinsic apoptosis, ginsenosides can engage an ER-stress axis that sensitizes cells to programmed death and connects upstream stress responses to both extrinsic and intrinsic cascades. For example (20 S)-Protopanaxadiol (PPD) has been reported to induce endoplasmic reticulum (ER) stress and activate the unfolded protein response (UPR) through PKR-like endoplasmic reticulum kinase (PERK)/eukaryotic translation initiation factor 2 alpha (eIF2α) phosphorylation and X-box binding protein 1 (XBP1) splicing, accompanied by apoptosis and changes including death receptor 5 (DR5) and caspase-8/9/3 activation, poly (ADP-ribose) polymerase (PARP) cleavage, and concomitant modulation of B-cell lymphoma 2 (Bcl-2) family members and mitochondrial potential. (Zhu et al., 2011). Consistently, CK has also been linked to ER-stress-associated apoptosis by suppressing p-STAT3 and enhancing ER stress markers; moreover, STAT3 knockdown has been reported to increase CK-induced ER stress and apoptosis (Zhang et al., 2018a). These findings are consistent with a role for ER stress as an upstream modulatory event that may lower the threshold for apoptosis in HCC-relevant contexts.

Some ginsenosides further increase apoptotic susceptibility. In particular, Rh2 has been reported to sensitize non-responsive HCC cells to TNF-related apoptosis-inducing ligand (TRAIL)-induced apoptosis, along with changes including upregulation of death receptor 5 (DR5), decreased X-linked inhibitor of apoptosis protein (XIAP) and survivin, and increased downstream caspase activity (Liu et al., 2022). Apoptosis induction is also influenced by the autophagy–apoptosis balance. Rk1 has been reported to induce autophagy early after treatment, where autophagy may partially serve as a survival response; importantly, pharmacologic or genetic autophagy inhibition is reported to enhance Rk1-driven apoptosis (Ko et al., 2009). Beyond this compensatory role, Rk1 has also been reported to be associated with changes consistent with autophagy-dependent apoptosis through AMP-activated protein kinase (AMPK)/mechanistic target of rapamycin (mTOR) signaling, characterized by increased microtubule-associated protein 1A/1 B-light chain 3-II (LC3-II) alongside reduced Bcl-2, which is consistent with autophagy flux being associated with caspase-mediated cell death (Wu et al., 2024).

Collectively, these mechanisms are reported in conjunction with measurable anti-tumor effects, including caspase activation, mitochondrial dysfunction, ER-stress–associated death programs, and sensitization of extrinsic apoptotic signaling. For instance, Rg3 has been reported to induce apoptosis and inhibit angiogenesis in orthotopic HCC models, with tumor shrinkage and improved survival reported (Hu et al., 2019). Likewise, CK and related metabolites have been reported to suppress proliferation and promote apoptosis through mitochondria- and/or ER-stress–linked caspase cascades (Zheng et al., 2014; Zhang et al., 2018a).

### 3.3 Autophagy and autophagic flux regulation by ginsenosides

Ginsenosides have been reported to regulate autophagy in a context-dependent manner in HCC models. Autophagy has been proposed to provide a cytoprotective stress-adaptation response in tumor cells; however, when autophagy signaling is reprogrammed and coupled with pro-death checkpoints, autophagy is reported to be associated with lethal autophagy-associated death outcomes, which are associated with suppression of tumor progression.

Ginsenoside Rk3 has been proposed to be associated with a shift from cytoprotective to pro-death autophagy outcomes. In HCC models, Rk3 has been reported to induce both autophagy and apoptosis, while being associated with inhibited proliferation. Mechanistically, Rk3 has been reported to induce G1 cell-cycle arrest and to be associated with autophagy–apoptosis coupling through the phosphoinositide 3-kinase (PI3K)/protein kinase B (AKT) pathway; proteomics and siRNA-based validation are consistent with functional modulation of PI3K/AKT by Rk3, and the overall phenotype is associated with reduced tumor growth *in vitro* and *in vivo* (Qu et al., 2023). In contrast, ginsenoside Rk1 has been reported to be associated with an early stage–dependent cytoprotective effect of autophagy. Rk1 has been reported to induce G1 arrest and autophagy at early time points, whereas apoptosis is not immediate. Importantly, pharmacologic or genetic inhibition of autophagy (e.g., bafilomycin A1 and beclin one silencing) is reported to enhance the anti-tumor efficacy of Rk1, consistent with early autophagy functioning as a survival mechanism (Ko et al., 2009). In a DEN + CCl4 primary HCC model, Rk1 is reported to further activate AMPK, suppress mTOR, increase LC3-II, and decrease Bcl-2, and is reported to be associated with caspase cascade activation, consistent with an autophagy-dependent apoptotic outcome in the tested setting (Wu et al., 2024). Beyond regulating tumor-intrinsic stress responses, autophagic flux has also been linked to therapy sensitivity. Rh2 suppresses HCC growth while promoting autophagy and inhibiting β-catenin signaling; importantly, interference with autophagy-related 7 (Atg7) or β-catenin abrogates the effects of Rh2, suggesting that autophagy may be necessary to disrupt tumor-maintaining signaling states (Yang et al., 2016). Conversely, Rg3 has been described as an autophagy-flow inhibitor: it blocks late-stage autophagy and prevents protective autophagy induced by doxorubicin, which is associated with synergy with doxorubicin in xenograft models while showing limited toxicity in normal liver cells (Kim et al., 2014). Overall, these findings are consistent with an integrative framework in which ginsenosides have been reported to suppress HCC in conjunction with (i) pathway-directed autophagy associated with apoptosis, (ii) context-dependent reprogramming of autophagy associated with lethal outcomes, and (iii) inhibition of autophagic flux that might otherwise contribute to chemotherapy resistance.

### 3.4 Mitochondrial dysfunction and mitochondria-driven apoptosis induced by ginsenosides

In hepatoma cells, ginsenoside effects have been reported to be associated with convergence on a shared upstream convergence point, namely, mitochondrial destabilization associated with redox imbalance. This mitochondrial disturbance is associated with promotion of the intrinsic apoptotic program, with caspase activation and increased apoptosis observed. In Hep3B cells, KRG extract and its major constituents Rg3 and Rh2 were shown to reduce cell viability while disrupting mitochondrial membrane potential, accompanied by elevated intracellular ROS and LDH release. Mechanistically, ROS accumulation has been reported alongside mitochondrial cytochrome c release, Bax/Bcl-2 shifting, and caspase-3 activation, which is consistent with mitochondria-centered apoptosis in the studied settings (Park et al., 2012). This intrinsic program is further supported by evidence that Rh2 has been reported to rapidly increase Bak/Bax translocation, with reports of cytochrome c release and downstream caspase activation observed. In addition, caspase-8 has been reported to participate in an amplification-like process in the Rh2-triggered apoptotic cascade, consistent with coordinated upstream mitochondria-dependent events and downstream caspase processing (Guo et al., 2012). Together, these preclinical observations are consistent with a mitochondria-centered model in which ginsenosides are reported to induce mitochondrial dysfunction, including mitochondrial outer membrane permeabilization (MOMP)-associated events, increased ROS, and loss of mitochondrial membrane potential, with reports of cytochrome c–dependent caspase activation and apoptosis execution observed in these studies.

Beyond Rg3/Rh2, other ginsenosides have been reported to be associated with reinforcing the mitochondrial switch in association with impaired antioxidant buffering. For example, Rg5 has been reported to target the antioxidant enzyme peroxiredoxin 1 (PRDX1), with reports of disrupted ROS detoxification, increased mitochondrial ROS accumulation, and intrinsic apoptosis being observed in HepG2 cells (Ye et al., 2026). Notably, mitochondrial apoptosis has also been reported to be associated with integration with death-receptor inputs. CK has been reported to induce mitochondrial membrane potential collapse and DNA damage, to increase Fas cell surface death receptor (Fas)/Fas ligand (FasL) signaling and the Bax/Bcl-2 ratio, to suppress pro-caspase-9/-3, and to inhibit Akt phosphorylation, which is consistent with a coupled Fas–mitochondria caspase-independent death route in the investigated HCC context (Zheng et al., 2014). Finally, Rg3 has been described to increase apoptotic rates in HCC cell lines in association with caspase-3 and Bax upregulation and Bcl-2 downregulation, reinforcing the end-of-chain mitochondrial caspase-dependent outcome (Zhang et al., 2012). Collectively, these preclinical observations are consistent with a mitochondria-centered mechanism for ginsenoside-induced apoptosis in liver tumor–relevant cell systems, with pathway differences emerging at the level of upstream redox control (e.g., PRDX1 inhibition) and signaling integration (e.g., Fas–mitochondria coupling).

### 3.5 Metabolic reprogramming through ginsenoside signaling

Ginsenosides have been reported to be associated with suppression of HCC, including effects on cell-death programs and reprogramming of metabolic pathways that are associated with tumor growth. Reported preclinical evidence is consistent with a metabolic shift in which ginsenosides have been reported to weaken glucose/energy supply and to reshape lipid metabolism associated with rapid proliferation and signaling.

In aerobic glycolysis–dependent contexts, ginsenoside Rg3 has been shown to inhibit glycolytic output and proliferation in association with the human microRNA-139–5p (hsa-miR-139–5p)/aurora kinase A (AURKA) axis. Rg3 has been reported to be associated with changes including elevated miR-139–5p, targeting of AURKA, reduced expression of glucose transporter 1 (GLUT1) and glucose transporter 4 (GLUT4), limited lactate/glucose secretion, increased apoptosis, and reversal of glycolysis-associated tumor expansion, with consistent findings reported in transplanted tumor experiments (Wei et al., 2025). Under hypoxic stress, CK has been described as rewiring glycolysis in association with suppression of BCL2-associated transcription factor 1 (Bclaf1), which is associated with reduced hypoxia-inducible factor 1-alpha (HIF-1α)–mediated glycolysis. In hypoxia models, CK reduces glycolysis-associated enzyme activity and real-time ATP production, decreases tumor glucose uptake *in vivo*, and restrains liver cancer cell proliferation (Zhang et al., 2020). CK has been reported to be associated with reinforcing inhibition of glycolysis-linked survival programs via protein kinase B (AKT)/mechanistic target of rapamycin (mTOR)/c-Myc signaling, with reductions in hexokinase-2 (HK2) and pyruvate kinase M2 (PKM2) abundance and suppression of glycolysis-linked survival programs, accompanied by apoptosis in HCC cell models (Shin et al., 2021). Together, these findings are reported to be associated with reduced anabolic drive and impaired cell-cycle progression.

Beyond glucose metabolism, ginsenosides have also been linked to lipid metabolism reprogramming, which can influence membrane biosynthesis and downstream signaling. In a therapeutic regimen context, Rg3 combined with cantharidin was reported to target the protein arginine methyltransferase 1 (PRMT1)–sterol regulatory element-binding transcription factor 1 (SREBF1) axis to modulate lipid metabolism and survival signaling, suppressing disease progression while mitigating hepatotoxicity in the tested model; given the combinational design, these effects should be interpreted as reflecting the tested regimen rather than the action of free Rg3 alone (Wang et al., 2026). Overall, preclinical evidence supports a coherent model in which ginsenoside-related signaling rewires glycolysis and lipid metabolism, collapses the energetic/biosynthetic foundation for tumor growth, and manifests as reduced proliferation with increased apoptosis and decreased tumor burden.

### 3.6 Gene expression and transcriptional control mediated by ginsenosides

Ginsenosides have been reported to suppress HCC by reprogramming gene expression at transcriptional and post-transcriptional levels, thereby shifting cellular states toward apoptosis- and arrest-permissive programs. A representative example is Rh2-mediated microRNA regulation. In HepG2 cells, Rh2 increased miR-146a-5p, and miR-146a-5p overexpression strengthened Rh2-induced inhibition of cell survival and colony formation, whereas miR-146a-5p inhibition weakened these effects, supporting a functional causal role for miR-146a-5p in Rh2 activity (Chen et al., 2018). In another miRNA-centered axis, Rh2 was also reported to upregulate miR-491, which negatively regulates EGFR at the translational level; importantly, restoring epidermal growth factor receptor (EGFR) signaling with recombinant EGFR abolished the anti-tumor effect, linking miRNA-mediated gene repression to reduced proliferation and viability in HCC models (Chen and Qiu, 2015). These gene-expression effects further intersect with metabolic regulation. For instance, Rg3 inhibits aerobic glycolysis through the hsa-miR-139–5p/AURKA axis: by targeting AURKA, Rg3 downregulates glycolytic executors including GLUT1/GLUT4, reduces lactate/glucose output, and promotes apoptosis while reversing glycolytic activation (Wei et al., 2025). Consistent with these pathway-level observations, transcriptomic profiling of Rh2-treated HepG2 cells indicated that transcriptional shifts converge on p53 signaling and DNA replication-related gene networks, aligning with the observed apoptosis-related marker changes (Zhang et al., 2019).

At the genome-regulatory level, ginsenoside Rg3 further provides an epigenetic mechanism involving DNA methylation. In human hepatocarcinoma cells, the (20 S)-Rg3 was shown to reduce global and promoter-region DNA methylation, accompanied by downregulation of DNA methyltransferase 3A (DNMT3a)/DNA methyltransferase 3B (DNMT3b) and altered methylation of tumor protein p53 (P53), B-cell lymphoma 2 (BCL2), and vascular endothelial growth factor (VEGF) (Teng et al., 2017). Together, the available preclinical evidence supports a model in which ginsenoside-driven miRNA repression, transcriptional remodeling, and DNA methylation changes are consistent with a shared contribution to apoptosis induction and growth suppression, accompanied by coordinated downshifts in signaling and survival programs in HCC cell models.

### 3.7 Cell cycle arrest by ginsenosides

In HCC-relevant settings, ginsenosides have been reported to reduce tumor cell proliferation by arresting cell-cycle progression at specific checkpoints, which can subsequently bias cells toward apoptosis. Preclinical evidence suggests that this effect is implemented through coordinated modulation of cyclin/CDK regulatory circuits as well as transcriptional and epigenetic control mechanisms.

A representative example is ginsenoside Rs4, which concurrently induces apoptosis and establishes a G1/S boundary arrest in SK-HEP-1 cells. In that model, Rs4 elevates p53 and p21^WAF1 (cyclin-dependent kinase inhibitor 1A, CDKN1A; also known as p21^WAF1) while downregulating cyclin E− and cyclin A-dependent kinase activities, and the arrested phenotype is accompanied by apoptosis markers such as DNA fragmentation and a detectable apoptotic peak (Kim et al., 1999). Consistent with checkpoint enforcement through cyclin/CDK control, ginsenoside Rg5 was reported to block G1/S transition in SK-HEP-1 cells by suppressing cyclin E–dependent kinase activity (cyclin E–CDK2)—associated with increased p21^Cip1/WAF1 (cyclin-dependent kinase inhibitor 1A; CDKN1A) and decreased cyclin E, along with reduced levels of CDK2 (cyclin-dependent kinase 2) and CDC25A (cell division cycle 25A) (Lee et al., 1997). Importantly, the direction of cell-cycle modulation may be stereospecific and pathway-selective rather than uniformly suppressive across all ginsenosides. Notably, in the same hepatoma cell context, Rg1 was reported to show the opposite trend by stimulating proliferation—upregulating cyclin E/CDK2 components and enhancing cyclin E–dependent kinase activity (Lee and Lee, 1996). These findings therefore support a model in which ginsenosides can exert checkpoint effects in a compound-specific manner, and not all family members necessarily converge on proliferation blockade.

Beyond cyclin/CDK proteins, some Rh2/Rg3-related mechanisms link cell-cycle arrest to broader regulatory remodeling. Rh2 has been described as a histone deacetylase (HDAC) inhibitor, decreasing total HDAC activity, increasing histone acetylation, and shifting cell-cycle distribution toward G0/G1 accumulation, with increased apoptosis in HepG2 cells (Qiang et al., 2021). Rh2 has also been linked to G0/G1 arrest via disruption of proliferative Wnt signaling: by activating glycogen synthase kinase-3 beta (GSK-3β) and promoting β-catenin degradation, Rh2 shifts downstream outputs toward pro-apoptotic programs (e.g., Bax) while reducing cyclin-associated and anti-apoptotic signals, ultimately suppressing proliferation and tumor progression (Shi et al., 2016). Additionally, Rh2 was reported to interfere with the heat shock protein 90 alpha–cell division cycle 37 (HSP90 A–Cdc37) chaperone system, leading to proteasome-mediated downregulation of cyclin-dependent kinase 4/6 (CDK4/6) (G0/G1 kinases) and CDK2 (G1/S kinase), thereby enforcing G0/G1 arrest and growth inhibition (Chen et al., 2021). At the epigenetic level, Rh2 was also reported to suppress oncogenic enhancer of zeste homolog 2 (EZH2), reduce histone H3 lysine 27 trimethylation (H3K27me3) at the cyclin-dependent kinase inhibitor 2A–2B (CDKN2A-2B) locus, and reactivate P14/P15/P16, resulting in G0/G1 arrest and reduced proliferation and migration phenotypes (Li et al., 2017).

Functionally, these cell-cycle checkpoint effects can translate into anti-tumor outcomes, including enhanced therapeutic responsiveness. For instance, in preclinical combination settings, Rg3 has been reported to reinforce growth suppression and apoptosis with chemotherapy (e.g., oxaliplatin) by downregulating proliferation-associated proteins such as PCNA and cyclin D1 (Shan et al., 2019). Moreover, cell-cycle dysregulation appears mechanistically coupled to death pathways in some models: panaxadiol-linked apoptosis has been associated with early changes in Cdk2 activity during mitochondrial dysfunction, providing a mechanistic bridge between “cell-cycle regulators” and “death execution” (Jin et al., 2003). Taken together, reported ginsenoside actions on cyclin/CDK control and upstream regulatory layers support a framework in which cell-cycle arrest contributes to reduced liver tumor cell viability and limits tumor growth, while often sensitizing cells to apoptosis in HCC-relevant preclinical systems.

### 3.8 Ferroptosis

In HCC, ginsenosides have been proposed to suppress tumor survival not only by activating apoptosis, but also by triggering ferroptosis, an iron-dependent form of regulated cell death driven by lipid peroxidation. Consistent ferroptosis-readouts include ROS accumulation, increased lipid peroxidation (e.g., elevated malondialdehyde (MDA)), glutathione (GSH) depletion, and ferrous iron (Fe^2+^) buildup, and these phenotypes can be largely reversed by ferroptosis inhibitors, supporting a ferroptosis-dependent mechanism.

For example, ginsenoside RK1 has been reported to inhibit HepG2 cell growth in a dose-dependent manner while promoting ferroptosis hallmarks, including reduced GSH and increased MDA/iron levels. Importantly, ferroptosis inhibitors substantially rescue the phenotype, and capacity for RK1-driven ferroptosis induction depends on ferroptosis suppressor protein 1 (FSP1), as supported by gain-/loss-of-function evidence (Jiang et al., 2024). In parallel, ginsenoside Rg1 has been shown to enhance ferroptosis sensitivity by downregulating solute carrier family seven member 11 (SLC7A11), thereby impairing HCC cell proliferation and migration (Wei et al., 2026). CK has also been linked to ferroptosis induction through stress-response transcriptional control. CK triggers ferroptosis in liver cancer cells via the forkhead box O (FOXO) pathway, accompanied by decreased phosphorylated FOXO1 (p-FOXO1) activity; in tumor models, CK suppresses growth while reducing SLC7A11 and glutathione peroxidase 4 (GPX4) expression (Chen et al., 2024). Mechanistically, multiple ginsenoside derivatives appear to converge on the same ferroptosis-protective nodes and lipid-redox execution circuits.

A key example is (20S)-protopanaxatriol ((20S)-APPT), which has been identified as a potent ferroptosis inducer by targeting FSP1. Inhibiting FSP1 promotes acyl-CoA synthetase long-chain family member 4 (ACSL4)-dependent arachidonic acid oxidation and mitochondrial ROS production; notably, combined inhibition of FSP1 and glutamate-cysteine ligase (GCS) further synergizes ferroptosis induction while sparing noncancerous cells, linking ferroptosis execution to both lipid-oxidation flux and redox supply (Liu et al., 2025). Consistent with this logic, CK has also been reported to amplify ferroptosis by undermining glutathione peroxidase 4 (GPX4). Specifically, CK inhibits the deubiquitinase OTU deubiquitinase 2 (OTUB2), which promotes ubiquitin-dependent GPX4 degradation, thereby strengthening ferroptosis and suppressing tumor growth in xenograft models (Jiang et al., 2026). Collectively, these preclinical studies support a coherent model in which ginsenosides modulate ferroptosis-suppressive nodes and ferroptosis-execution circuits—notably the FSP1/SLC7A11/FOXO/GPX4 axis and the iron–lipid ROS circuitry—thereby promoting ferroptosis-associated cell death and reducing HCC tumor burden.

Figure 1 schematically summarizes the major reported preclinical mechanisms by which ginsenosides (e.g., Rh2 and Rg3) may suppress HCC, including apoptosis/mitochondrial signaling, modulation of autophagy and cellular stress balance, cell-cycle arrest, and reprogramming of the tumor microenvironment/immune–inflammatory axis, with additional relevance to ferroptosis-related and epigenetic/transcriptional regulatory pathways.

**FIGURE 1:**
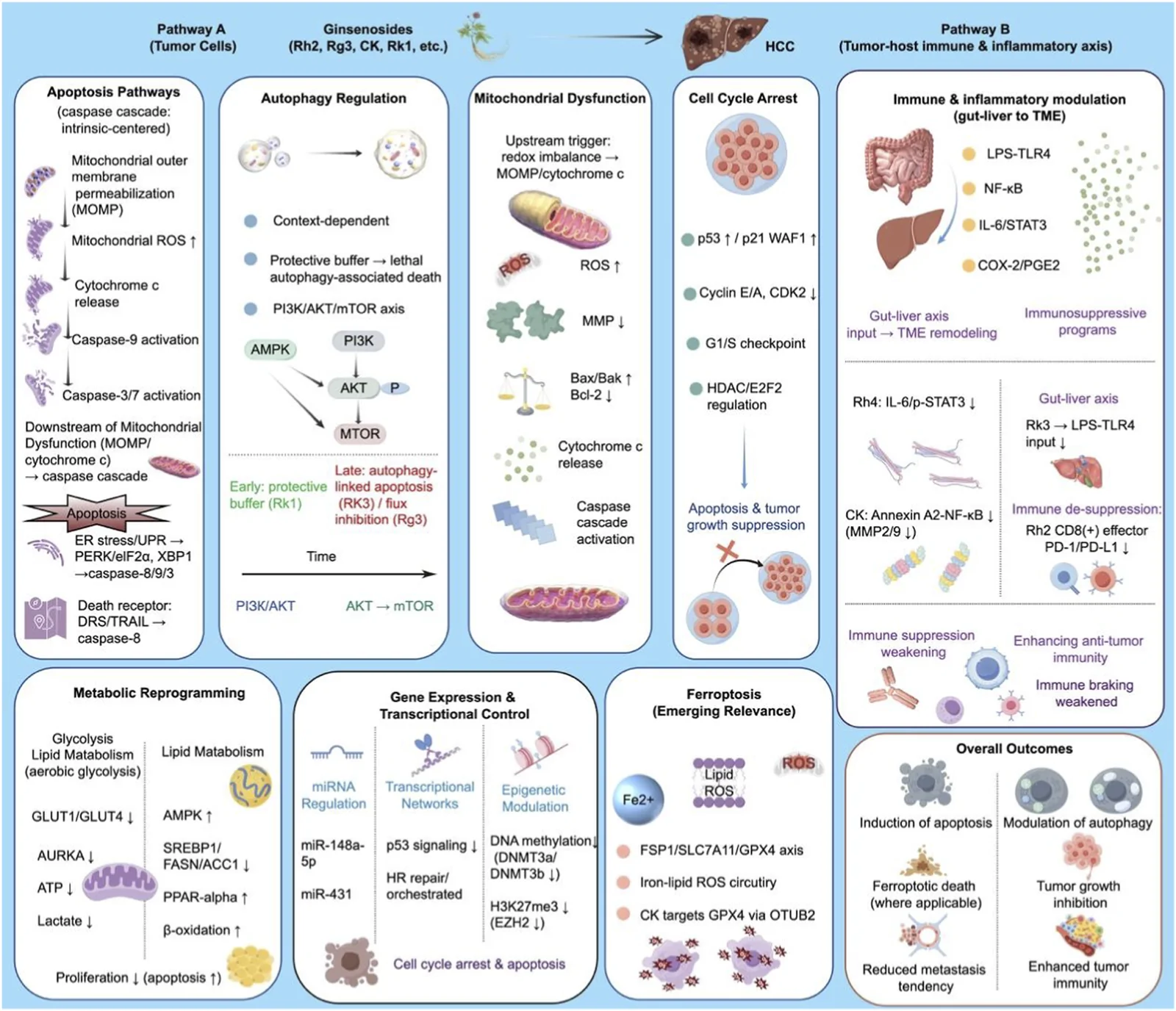
Proposed mechanisms of ginsenosides (Rh2, Rg3, CK, Rk1, *etc.*) in hepatocellular carcinoma (HCC). In tumor cells, ginsenosides may induce mitochondrial dysfunction and apoptosis, regulate autophagy in a context-dependent manner, and promote growth inhibition through cell-cycle arrest (e.g., via p53/p21 and cyclin/CDK signaling). They also modulate the tumor microenvironment and immune/inflammatory pathways by suppressing inflammatory signaling (e.g., LPS–TLR4/NF-κB, IL-6/STAT3, COX-2/PGE2) and reducing immunosuppressive programs (e.g., PD-1/PD-L1).

## 4 Clinical translation and therapeutic potential of ginsenosides against HCC

### 4.1 Ginsenosides as adjuncts to chemotherapy in HCC

From a clinical-translation perspective, ginsenosides are increasingly positioned not only as standalone anti-HCC agents, but also as adjuncts that may enhance the efficacy of established therapies and help counter treatment resistance. In preclinical cell models, combination regimens have shown stronger anti-tumor effects than either agent alone. For instance, regorafenib combined with a ginsenoside was associated with stronger suppression of HepG2 proliferation than monotherapy, together with lower survivin mRNA levels and a shift toward apoptosis-related signaling (Wang et al., 2020). Similarly, in SMMC-7721 cells, Rg3 plus oxaliplatin enhanced apoptosis and reduced proliferation-associated proteins, including proliferating cell nuclear antigen (PCNA) and cyclin D1 (Shan et al., 2019). These findings support a pathway-consistent and/or perturbation-supported hypothesis that adjunct ginsenosides may reduce therapy-driven survival/damage-tolerance programs in the tested systems; however, such preclinical mechanism–efficacy links should not be interpreted as established clinical causality.

Beyond platinum agents, Rg3 has been reported to inhibit late-stage autophagy and thereby sensitize HCC to doxorubicin, consistent with the possibility that autophagy may act as a protective response during chemotherapy (Kim et al., 2014). In the same translational rationale, ginsenoside Rd has been used to complement combretastatin A4 phosphate (CA4P), a vascular disrupting agent that induces hypoxia and elevates HIF-1α; in the cited experimental context, the CA4P/Rd regimen reduced HIF-1α via PI3K/AKT/mTOR inhibition and was associated with expanded necrosis and delayed tumor growth (Yang et al., 2021). Importantly, clinical evidence supports the adjunct concept at the level of patient-relevant outcomes: a 2024 meta-analysis of randomized controlled trials evaluating ginsenosides as adjuvant therapy in HCC reported improved disease control and objective response, better quality of life, higher 1- and 2-year overall survival rates, and fewer adverse events (e.g., reduced nausea/vomiting and myelosuppression) (Zhang et al., 2024). Overall, preclinical data motivate testable hypotheses that ginsenosides may amplify tumor cell death signals while attenuating therapy-limiting adaptive mechanisms, but clinical outcome claims should be attributed to the cited human evidence rather than inferred directly from mechanistic findings alone.

### 4.2 Ginsenosides as adjuvant agents for TACE

For patients with advanced HCC, TACE is used for regional disease control; however, its clinical benefit can be limited by hypoxia-driven angiogenesis and subsequent progression. In this context, ginsenoside—particularly Rg3—has been evaluated as an adjunct to improve outcomes and tolerability. In a single-center, open-label randomized controlled trial in patients with advanced HCC (BCLC stage C; Child–Pugh class A; ECOG 0–1), patients were randomly assigned in a 2:1 ratio to TACE plus oral ginsenoside Rg3 (*n* = 152) or TACE alone (*n* = 76); the trial was registered at the Chinese Clinical Trial Registry (ChiCTR-TRC-11001643) (62). Relative to TACE alone, the Rg3 combination arm showed longer overall survival (median 13.2 vs. 10.1 months; HR 0.63, 95% CI 0.46–0.85; *P* = 0.002) and a higher disease control rate (69.7% vs. 51.3%; *P* = 0.012) (62). Safety was assessed using NCI-CTCAE v3.0; Rg3-related adverse events occurred more frequently in the combination arm, including constipation (133/152 [87.5%] vs. 50/76 [65.8%]; grade 3–4: 2/152 [1.3%] vs. 0/76 [0%]) and epistaxis (8/152 [5.3%] vs. 0/76 [0%]; grade 3–4: 0/152 [0%] vs. 0/76 [0%]) (Zhou et al., 2016). In parallel, a preclinical orthotopic rat study reported that Rg3 combined with transarterial embolization reduced VEGF/VEGFR2-associated signaling and angiogenesis, and was associated with reduced tumor progression and improved survival versus embolization alone, supporting a pathway-consistent, hypothesis-generating mechanism in the experimental context rather than established causal clinical efficacy (Zhou et al., 2014). At the evidence level, meta-analyses of randomized trials indicate that TACE combined with ginsenosides may improve objective response rate, disease control rate, quality of life, and certain survival-related endpoints, while also reducing several treatment-related adverse events. However, effect magnitudes and safety estimates may vary across trials due to differences in study design and reporting consistency, warranting cautious interpretation (Zhang et al., 2024; Zhu et al., 2021).

### 4.3 Nano-formulations and nanomaterials to enhance the therapeutic efficacy of ginsenosides in HCC

During translational development for HCC, ginsenoside-based agents often face practical limitations, including poor aqueous solubility, limited *in vivo* stability, restricted bioavailability, and insufficient drug exposure at the tumor site. To address these bottlenecks, “formulation engineering” has increasingly been used to improve systemic circulation and liver/tumor accumulation, and to enable stimulus-responsive, on-site release. Because the majority of this evidence currently comes from preclinical models, these strategies should be interpreted as delivery- and feasibility-supported hypotheses for enhancing therapeutic performance, rather than as established clinical benefit.

One representative direction is imaging-guided theranostics using magnetic resonance imaging (MRI) contrast. Zhao et al. constructed a Fe@Fe (3)O (4) core–shell nanoparticle system (Nano-Fe-GSS) by immobilizing pre-activated ginsenoside onto the nanoparticle surface; in the experimental context, the formulation demonstrated enhanced MRI signal and supported a “trackable” delivery concept for tumor therapy (Zhao et al., 2018). Beyond imaging coupling, polymer- and lipid-based nanocarriers have also been explored to improve stability and tumor selectivity. For example, PLGA-PEG-Rg3 (Zheng et al.) showed stronger inhibition of HepG2 growth *in vitro* and improved anti-tumor activity and survival *in vivo* compared with free Rg3, consistent with improved bioavailability and delivery (Zheng et al., 2024). Similarly, pH-sensitive Rh2 liposomes (Sun et al.) improved drug release under acidic conditions and were associated with enhanced anti-tumor activity in liver cancer cell models, supporting the rationale for microenvironment-responsive delivery (Sun et al., 2025). In addition to “tumor-cell-level” effects, some studies have reported systemic remodeling associated with nanodelivery; Ren et al. reported that Rg3 nanoparticle conjugation was associated with suppression of HCC progression and lung metastasis and with changes in gut microbiota and metabolic networks in a tumor-bearing mouse model, suggesting a plausible delivery–microenvironment–linked therapeutic enhancement mechanism that warrants clinical validation (Ren et al., 2020). Targeting strategies and multi-component delivery have also been proposed; hyaluronic acid (HA)-modified nanostructured lipid carriers enabled enriched delivery via cluster of differentiation 44 (CD44)-related mechanisms and co-delivery of multiple components, showing improved tolerability and anti-tumor efficacy in preclinical evaluations (Sun et al., 2020).

Furthermore, nanomaterials have also been explored to expand non-traditional therapeutic capacities, including sonodynamic therapy (SDT). Yang et al. loaded Rk1 into manganese-doped hollow titania (Rk1@MHT), where manganese doping was designed to tune absorption and band structure to enhance ROS generation under ultrasound irradiation. In the reported experimental context, the formulation delivery was accompanied by modulation of glutathione synthesis-related steps, which reinforced intracellular oxidative stress, and the system also provided T1-weighted MRI functionality; in tumor-bearing mice, greater liver-tumor clearance was observed (Yang et al., 2023b). From the perspective of improving “uptake” and drug accessibility, other ginsenoside derivatives have likewise been formulated into nanocarriers. For instance, Zhang et al. prepared chitosan nanoparticles loaded with CK (CK-NPs); *in vitro*, improved CK water solubility and enhanced cellular uptake were associated with stronger cytotoxicity and apoptosis-related phenotypes in HepG2 cells (Zhang et al., 2018b). Regarding alternative administration routes, Sargazi et al. employed alginate/PLGA nanoparticles for oral delivery of GRg3; the study emphasized potential benefits including gastrointestinal stability, sustained release behavior, and supportive *in vivo* safety and antioxidant-related outcomes, thereby offering translational feasibility signals that still require clinical confirmation (Sargazi et al., 2026). More refined release control has also been pursued with stimulus-responsive systems. Xu et al. developed a dual-responsive Rh2 nanodelivery platform responsive to both pH and redox conditions (GSH), where release was triggered under acidic and reducing milieus and higher cytotoxicity was observed in HepG2 cells compared with free Rh2 (Xu et al., 2020). Mechanistic observations in this preclinical setting included increased ROS, reduced antioxidant-related indices, and decreased mitochondrial membrane potential; these results are consistent with an oxidative-stress-associated mitochondrial apoptosis phenotype, but they should be interpreted as mechanistic support within the experimental model rather than definitive causal proof.

Overall, current preclinical evidence suggests that nanomedicine strategies may improve practical usability of ginsenosides in HCC by enhancing delivery efficiency, potentially increasing tumor exposure and enabling more controllable release and therapeutic modalities, with reported tolerability signals. However, differences in nanocarrier composition, administered dosing/exposure, and the limited availability of standardized long-term toxicological data across studies highlight the need for further validation to support more robust clinical translation.

### 4.4 Ginsenosides in combination strategies with conventional therapies and targeted agents

Combination strategies are being explored to strengthen the antitumor efficacy of ginsenosides while addressing limitations of single-agent treatment. At present, most supportive evidence remains preclinical (cell- and animal-based), where observed synergy and biomarker modulation should be interpreted as hypothesis-generating or context-consistent rather than clinically validated causality. Mechanistically, reported ginsenoside combinations in HCC models have been associated with coordinated regulation of oncogenic signaling, metabolic reprogramming, inflammatory/immune-related pathways, and cell-fate responses. For example, *in vitro* data indicate that ginsenoside Rg3 combined with cantharidin (CTD) produces synergistic inhibition of HCC cell proliferation, migration, and invasion, outperforming either agent alone. In the same experimental framework, the antitumor phenotype was attributed to dual modulation of lipid metabolism–associated signaling (including PI3K/AKT/mTOR pathway activity) and protein arginine methyltransferase 1 (PRMT1)-mediated epigenetic regulation, and a PRMT1–sterol regulatory element-binding transcription factor 1 (SREBF1) relationship was described based on the study’s network pharmacology/molecular interaction analyses. In addition, co-treatment with Rg3 reportedly mitigated CTD-associated hepatotoxicity *in vivo*, reflected by preservation of serum alanine aminotransferase (ALT)/aspartate aminotransferase (AST) levels and pathway-level trends consistent with reduced oxidative stress and improved metabolic homeostasis within that animal model (Wang et al., 2026). Overall, these findings support the plausibility of Rg3 as a combination partner, while the mechanistic links should be treated as perturbation- and pathway-consistent evidence confined to the cited preclinical system.

Beyond metabolic regulation, curcumin and total ginsenosides (TG) have also been reported to show synergistic anti-liver-cancer effects with immunomodulatory and anti-inflammatory characteristics in HCC mouse models. Specifically, the combination was associated with reduced tumor growth and with modulation of immune escape–related signaling (e.g., programmed death-ligand 1 (PD-L1)) as well as inhibition of the Toll-like receptor 4 (TLR4)/nuclear factor kappa-light-chain-enhancer of activated B cells (NF-κB)–centered inflammatory cascade; downstream changes included markers linked to inflammatory and tumor progression programs (inducible nitric oxide synthase (iNOS) and matrix metalloproteinase 9 (MMP9)) (Deng et al., 2020). These observations indicate pathway-consistent immune/inflammatory reprogramming in that model, but they do not establish that the proposed mechanisms are the validated drivers of clinical benefit. In another combination line emphasizing cell-fate regulation, Rh2 combined with luteolin has been reported to suppress HCC cell proliferation, migration, and invasion and to induce cellular senescence. Mechanistically, the study reported attenuation of the nuclear factor erythroid 2–related factor 2 (Nrf2)/heme oxygenase-1 (HO-1) pathway and activation of tumor protein p53 (p53)/cyclin-dependent kinase inhibitor 1 A (p21), and it included rescue evidence using an Nrf2 activator, which—within that experimental context—supports a perturbation-influenced causal role of the Nrf2/HO-1 axis in the observed senescence phenotype. *In vivo*, tumor growth inhibition was reported with no major organ pathological abnormalities in the cited model (Huang et al., 2026). Finally, network pharmacology and bioinformatics analyses have been used to support a multi-target/multi-pathway rationale for CTD/Rg3 combinations. Reported pathway enrichment patterns implicated metabolic pathways alongside immune-related processes, and target screening highlighted candidates such as aldo-keto reductase family one member D1 (AKR1D1), solute carrier family 16 member 4 (SLC16A4), and aldehyde dehydrogenase three family member B1 (ALDH3B1), while docking analyses suggested high-affinity binding to ALDH3B1, a molecule discussed in relation to immune microenvironment remodeling (Gou et al., 2026). Because these elements are largely computational/associative, they should be viewed as association-level or target-prioritization evidence, requiring experimental validation for direct mechanistic causality. Overall, the current literature suggests that ginsenoside-based combination strategies can enhance antitumor activity and reshape tumor-relevant signaling in experimental HCC settings. However, differences in combination composition, dosing/exposure, experimental tumor models, and the extent of longitudinal safety evaluation mean that these findings should be interpreted as preclinical opportunities until supported by controlled clinical trials and interaction/safety assessment in patients.

As summarized in Figure 2, ginsenoside-based strategies for HCC can be organized into (i) adjunct chemotherapy/TACE, (ii) nano-formulation and nanomaterials for delivery, and (iii) combination therapies with conventional/targeted agents. Importantly, Figure 2 distinguishes clinical evidence (human-associated outcomes and selected adverse-event profiles) from preclinical evidence (mechanistic findings in cell/animal models), to avoid inferring direct clinical causality from pathway modulation. Mechanistically, the preclinical literature most consistently supports effects on survival/apoptotic signaling, stress-related cross-talk (e.g., ROS/ER stress), and hypoxia-driven resistance programs (e.g., VEGF/VEGFR2-linked signaling), while combination regimens and delivery platforms may further influence inflammation/immune checkpoint–related pathways (e.g., TLR4/NF-κB and PD-L1). These mechanistic links are interpreted through an evidence-tier framework described in the main text, where mechanistic observations are treated as hypothesis-generating until validated by controlled clinical studies.

**FIGURE 2:**
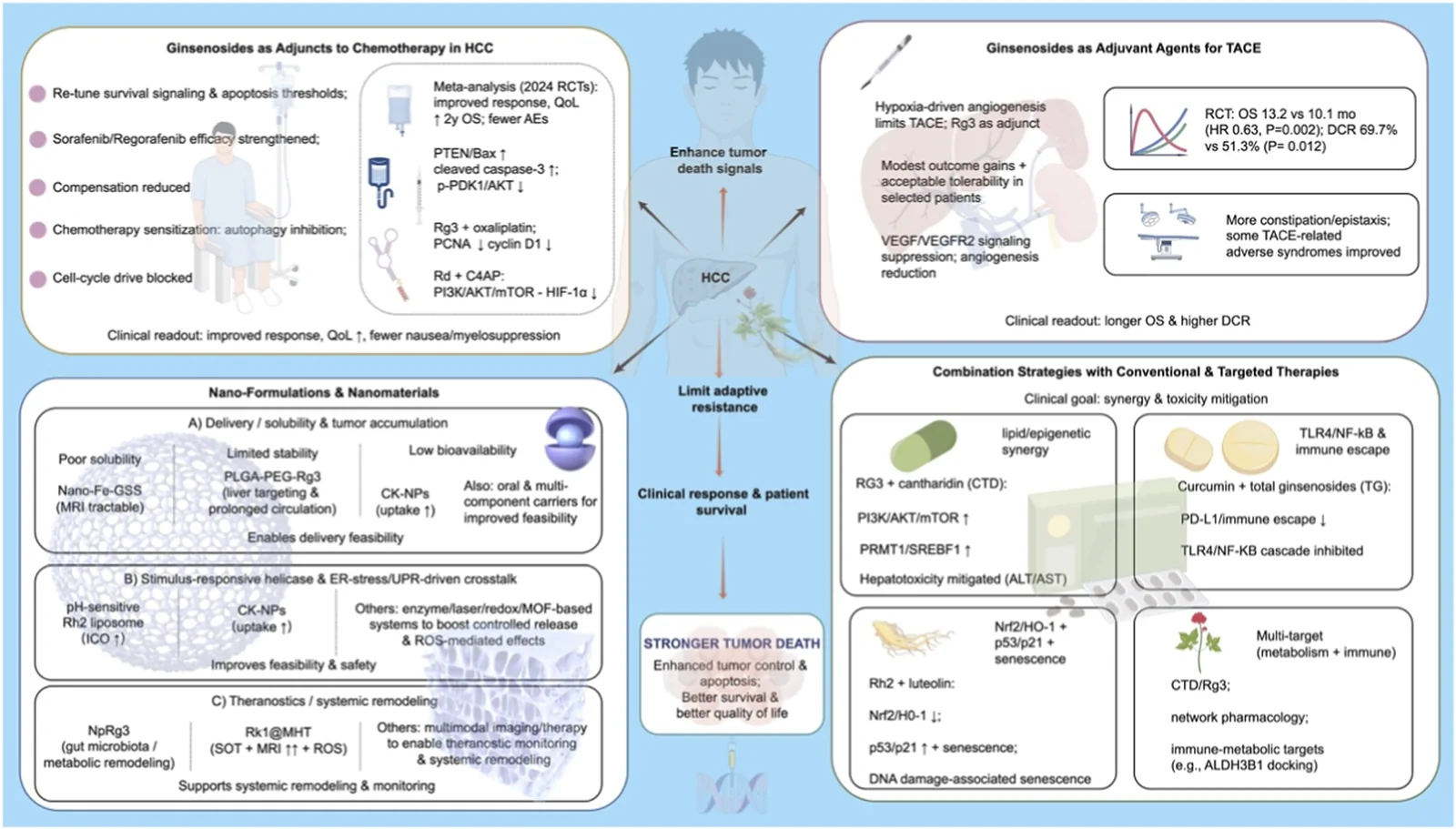
Overview of ginsenoside-based strategies for hepatocellular carcinoma (HCC), integrating adjunct chemotherapy/TACE, nano-formulation and nanomaterials, and combination therapies with conventional/targeted agents. The schematic distinguishes evidence tiers by summarizing preclinical evidence (cell/animal mechanistic associations) and human evidence (reported outcomes such as OS/QoL and selected AEs) separately, where available, from the included studies. This figure is intended to illustrate translational rationale rather than establish direct clinical causality from mechanistic findings.

## 5 Conclusion and future perspectives

This review summarizes preclinical evidence suggesting that ginsenosides—particularly Rh2 and Rg3—may target multiple pathways to produce anti–HCC effects. The antitumor activities discussed are reported to enhance apoptosis (including mitochondrial/caspase-related signaling), modulation of autophagy and cellular stress balance, and reprogramming of the tumor microenvironment, with additional coverage of emerging relevance to ferroptosis-related regulation. Beyond *in vitro* findings, the review highlights promising results in animal models and frames translational potential through strategies such as combination therapy (e.g., as an adjunct to treatments like TACE) and nano-delivery systems to improve solubility, stability, and tumor exposure.

Despite these strengths, the current evidence base has important limitations. First, clinical validation remains limited and heterogeneous, with inconsistent study design elements across reports (e.g., dose, dosing schedule, and endpoints), which reduces comparability. Second, mechanism-focused conclusions frequently rely on correlative observations and therefore require stronger causal support to confirm the involvement of specific pathways. Third, translation is constrained by issues common to natural products, including pharmacokinetics, bioavailability, safety/tolerability, and manufacturing and standardization challenges. Although existing studies provide supportive mechanistic insights, translational reliability is further limited by the predominance of certain *in vivo* models. In particular, most reported *in vivo* evidence derives from subcutaneous xenograft models, which do not recapitulate the orthotopic hepatic microenvironment of liver cancer, including tumor–stroma interactions, local metabolic cues, and the physiological immune context. Consequently, conclusions related to immunomodulatory effects—especially those inferred from immune checkpoint or cytokine readouts—may have limited generalizability to clinically relevant liver tumor settings.

Future studies should therefore validate key mechanistic and therapeutic claims using orthotopic tumor models and, where feasible, genetically engineered mouse models (GEMMs) that better reflect liver-specific architecture, microenvironmental signals, and immune complexity. In addition, future experimental designs should incorporate more clinically aligned endpoints and adopt standardized pharmacokinetics (PK)/pharmacodynamics (PD) assessment and dosing schedules to improve cross-study comparability. Finally, translational progress would be strengthened by prioritizing well-designed, adequately powered clinical trials with patient stratification and robust long-term outcomes, conducting pathway-specific causal mechanism studies using genetic or pharmacologic interference, and advancing delivery platforms with integrated evaluations of efficacy, safety, immune compatibility, and scalability.
