# Supplementary material for: Ginsenosides in hepatocellular carcinoma: preclinical evidence, mechanisms, and clinical implications
Source: Front Pharmacol. 2026 Oct 22;17:1973780. doi: 10.3389/fphar.2026.1973780 (PMC13736101; doi:10.3389/fphar.2026.1973780)
Supplement: Supplementary file 1 [file Supplementaryfile1.docx]

**Supplementary figure 1 Flow diagram of literature identification, screening, full-text eligibility assessment, and study inclusion.**

**Supplementary table 1 Preclinical studies of ginsenoside Rh2 in hepatocellular carcinoma (HCC) models.**

| **Ginsenoside** | **Model** | **Dose, time, and route** | **Results** | **Mechanistic implication** | **Reference** |
| --- | --- | --- | --- | --- | --- |
| Rh2 | SK-HEP-1 | 7.5 mg/mL | Apoptosis ↑ (DNA fragmentation/flow cytometry/morphology consistent with apoptosis) | Caspase-dependent apoptosis, supported by caspase-3 activation and PARP cleavage; apoptosis was Bcl-2-insensitive. | ^1^ |
| Rh2 | SK-HEP-1 | 12 µM | Apoptosis ↑ | Bax/Bak translocation–associated mitochondrial cytochrome c release and caspase activation; siRNA experiments supported a functional contribution of caspase-8 to caspase-9 activation and apoptosis. | ^2^ |
| Rh2 | Hep3B | 10 µM | Apoptosis ↑; cell viability ↓ | ROS-associated mitochondrial apoptotic signaling, accompanied by reduced mitochondrial membrane potential, cytochrome c release, Bax/caspase-3 activation, and Bcl-2 downregulation. | ^3^ |
| Rh2 | HepG2 | 10–160 µM | Migration ability ↓ (Transwell-based) | Reduced migration associated with HDAC4 upregulation, suppression of AP-1 transcriptional activity, and decreased MMP3 expression. | ^4^ |
| Rh2 | SMMC-7721and HepG2 cells; SMMC-7721 subcutaneous xenografts in male NOD/SCID mice | 20 µM for 24–96 h; 1 mg/kg, intravenous tail-vein injection twice weekly for 1 month. | Cell proliferation/survival ↓; animal tumor growth inhibition observed | miR-491 upregulation–associated EGFR suppression; recombinant EGFR reversed the antitumor effect, supporting a functional role for the miR-491/EGFR axis. | ^5^ |
| (20R)-Rh2; (20S)-Rh2 | Female H22 hepatoma-bearing mice | 2, 3, 4, or 6 mg/kg by oral gavage for 10 days | Tumor growth ↓; inhibition rate up to **46.8% (20R)** and **42.2% (20S)**; apoptosis index **3.87% (20R)** and **3.80% (20S)** | In vivo tumor-growth inhibition associated with increased tumor-cell apoptosis and reduced Bcl-2 mRNA expression. | ^6^ |
| (20S)-Rh2 | HepG2; HepG2 xenograft in BALB/c nude mice | 50-150 μM for 24–72 h; 20 mg/kg by oral gavage once daily; 20days | Cell cycle arrest (G0/G1) + early apoptosis ↑; in vivo: tumor burden/weight ↓ | GSK-3β activation–associated β-catenin downregulation; β-catenin overexpression attenuated Rh2 activity, supporting a functional role for the GSK-3β/β-catenin axis. | ^7^ |
| Rh2 | HepG2 and Huh7 cells; NOD/SCID mice bearing subcutaneous HepG2 xenografts | 0.01-1mg/mL; 1 mg/kg by tail-vein injection twice weekly for 4 weeks | Cell viability ↓ (dose-dependent); CSC-like cells ↓; tumor sphere formation ↓ | Autophagy induction and β-catenin signaling inhibition; Atg7 knockdown and β-catenin overexpression abolished Rh2 effects, supporting a functional role for autophagy/β-catenin crosstalk. | ^8^ |
| (20S)-Rh2 | HepG2 and Hep3B cells; BALB/C mice bearing subcutaneous HepG2 xenografts | 10-80µM for 12-72h  ; 5 mg/kg by intraperitoneal injection, 5 weeks | Proliferation ↓; migration ↓; cell cycle arrest at G0/G1; in vivo: tumor growth ↓ | EZH2 downregulation–associated derepression of the CDKN2A-2B gene cluster (P14, P15, and P16) through reduced H3K27me3; siRNA experiments supported a functional role for EZH2. | ^9^ |
| Rh2 | HepG2, Huh7 and SMMC-7721 cells; nude mice bearing subcutaneous HepG2 xenografts | 20 µg/ml 20 µg/mL for 48 h; 1 mg/kg by tail-vein injection twice weekly, 4 weeks | Anti-proliferation ↑; apoptosis ↑; colony formation ↓ | miR-146a-5p upregulation; miR-146a-5p overexpression enhanced, whereas its inhibition attenuated, Rh2-induced anti-proliferative and pro-apoptotic effects, supporting a functional role for miR-146a-5p. | ^10^ |
| Rh2 | HepG2 | **20 μg/ml** | lncRNA/mRNA expression profile consistent with Rh2 treatment | Rh2-associated global lncRNA/mRNA expression changes; the functional relevance of individual transcripts was not validated. | ^11^ |
| (20S)-Rh2 | HepG2 | 20/50 μM | Proliferation ↓; apoptosis ↑ | Transcriptomic changes consistent with modulation of p53 signaling and DNA-replication pathways, accompanied by increased caspase-3, caspase-6, and PARP expression; functional roles of individual genes were not validated. | ^12^ |
| (20S)-Rh2 | HepG2, SK-HEP-1 | 20 μg/ml | Cell survival/proliferation ↓; cell cycle arrest at G0/G1 | HSP90A target engagement supported by thermal-shift analysis, associated with disruption of the HSP90A–Cdc37 complex and proteasome-mediated downregulation of CDK2/4/6. | ^13^ |
| (20S)-Rh2 | HepG2; HepG2-derived xenografts | 80 µM for 6-48 h; 20 mg/kg by oral gavage once daily for 20 days | Cell cycle: G0/G1% ↑; apoptosis rate ↑; in vivo: CD31 ↓ and Ki-67 ↓ | Effects associated with reduced total HDAC activity, increased histone acetylation, and altered MAPK signaling (increased p-JNK and reduced p-ERK); no pathway-specific perturbation experiments were reported. | ^14^ |
| (20S)-Rh2 | HepG2 | 80 µmol/L; 20 mg/kg, 20days | HUVEC growth/migration ability ↓ (in co-culture) | Annexin A2–dependent inhibition of STAT3/VEGF signaling; the Annexin A2-K301A binding-deficient mutant abolished STAT3 inhibition, supporting a functional role for Annexin A2 target engagement. | ^15^ |
| (20S)-Rh2 | HepG2, SMMC-7701 and LM-3 cells; subcutaneous HepG2 xenografts female BALB/c nu/nu mice | 12.5-50 μM for 30 min; Rh2 (1 mg/kg) by intraperitoneal injection for 8 days | Cell viability ↓; apoptosis ↑; in vivo therapeutic efficacy enhanced | TRAIL sensitization associated with DR5 upregulation, XIAP and Survivin downregulation, and enhanced caspase cascade activation; pathway-specific perturbation experiments were not reported. | ^16^ |
| Rh2 | Huh-7; subcutaneous H22 tumors in female C57BL/6 mice | 5-12.5 μM for 48 h; 15 or 30 µmol/kg by oral gavage for 15 days | Invasion and metastasis ↓ (dose-dependent); in vivo metastasis burden ↓ | Inhibition of EMT associated with suppression of the c-Jun/COX2/PGE2 pathway; exogenous PGE2, LPS-mediated COX2 activation, and c-Jun overexpression reversed the anti-metastatic/anti-EMT effects, supporting a functional role for this pathway. | ^17^ |
| (20S)-Rh2 | HepG2 | 0-20 mg/L | HUVEC proliferation/migration/tube formation ↓; HepG2 proliferation ↓ and apoptosis ↑ | Anti-angiogenic effects associated with reduced VEGF and MMP-2 expression, together with suppression of GPC3-mediated Wnt/β-catenin signaling; GPC3 downregulation enhanced Rh2 effects, supporting a functional contribution of GPC3 to Rh2 activity. | ^18^ |
| Rh2 | Hepa1-6 | 0-40 µM | Bulk cell viability ↓; apoptosis ↑ in Hepa1-6; CD8(+) T cell effector features ↑; PD-L1/PD-1 ↓; Tregs ↓ | Immunomodulatory effects associated with enhanced CD8(+) T-cell degranulation and IFN-γ expression, reduced PD-L1/PD-1 expression, decreased TGF-β1, IL-6, and IL-10 levels, and reduced Treg frequency; causal contributions of these immune changes were not functionally validated. | ^19^ |

**Note:** HepG2 is generally considered a hepatoblastoma-derived cell line and is sometimes presented separately from established HCC models, although it is frequently used as an HCC-like in vitro model. Accordingly, studies using HepG2 were retained only as supportive liver-tumor–relevant evidence and were not treated as equivalent components of the main HCC evidence base, with limited transferability to established HCC settings. The identity and tumor origin of SK-HEP-1 cells have been debated; therefore, findings from this cell line should be interpreted cautiously. **Abbreviations** HCC = hepatocellular carcinoma; Rh2 = ginsenoside Rh2; SK-HEP-1, Hep3B, HepG2, SMMC-7721, Huh-7, H22 = human or mouse hepatoma cell lines / transplanted tumor models; HUVEC = human umbilical vein endothelial cells; CSC = cancer stem cell; G0/G1 = cell cycle phases; CD31 = platelet endothelial cell adhesion molecule 1; Ki-67 = proliferation marker; PD-L1 = programmed death-ligand 1; PD-1 = programmed death-1; Tregs = regulatory T cells; EMT = epithelial–mesenchymal transition.

**Supplementary Table 2. Preclinical studies of ginsenoside Rg3 in hepatocellular carcinoma (HCC) models.**

| **Ginsenoside** | **Model** | **Dose, time, and route** | **Results** | **Mechanistic implication** | | **Reference** |
| --- | --- | --- | --- | --- | --- | --- |
| **Rg3** | Hep1-6 and HepG2 cells; subcutaneous Hep1-6 tumor-bearing female C57BL/6 mice | 0-200 μg/mL for 0-48h; 3.0 mg/kg by intratumoral injection for 10 days | **Cells:** proliferation ↓; apoptosis ↑ (time-/concentration-dependent). **Animals:** tumor growth ↓; mouse survival time ↑ (in Rg3+CTX vs control; CTX combination also included). | Intrinsic mitochondrial apoptotic signaling, accompanied by mitochondrial membrane-potential loss, Bax upregulation, Bcl-2/Bcl-XL downregulation, and caspase-3 activation; caspase-3 inhibitor blockade supported a functional role for caspase activation. | ^20^ | |
| **Rg3** | SMMC-7721 and HepG2 cells | **0–100 μg/mL;** | proliferation inhibition rate ↑ (dose-/time-dependent); apoptosis rate ↑ (dose-/time-dependent). | Apoptosis associated with increased caspase-3 and Bax mRNA expression and decreased Bcl-2 mRNA expression, consistent with involvement of mitochondrial caspase-dependent apoptotic signaling; no mechanistic perturbation experiments were reported. | ^21^ | |
| **(20S)-** **Rg3** | HepG2, SK-Hep1, Huh-7, Hep3B; subcutaneous Huh-7 xenografts in male nude mice | 12.5–100 µM for 16 h; 20 mg/kg orally once daily, 21days | **Cells:** TRAIL-induced cell death/apoptosis ↑. **Animals:** therapeutic efficacy of TRAIL combination enhanced; tumor growth inhibition/benefit in xenograft model | TRAIL sensitization associated with CHOP-mediated transcriptional upregulation of DR5 and enhanced apoptosis. | ^22^ | |
| **Rg3** | Subcutaneous H22-bearing KM mice | 3 mg/kg intraperitoneally once daily, 10days | H22 tumor growth inhibited; inhibition rate **23.6% (20(S))** and **40.9% (20(R))**; (immune function enhanced as additional phenotype). | In vivo antitumor activity associated with enhanced ConA-induced lymphocyte proliferation and increased Th1-type cytokines (IL-2 and IFN-γ); the greater activity of (20R)-Rg3 than (20S)-Rg3 suggests stereospecific immunomodulatory effects, although causal immune mechanisms were not directly validated. | ^23^ | |
| **Rg3** | HepG2 | 178.03/326.84 µg/ml | cell growth inhibition ↑ (20(S) > 20(R)) | Stereospecific growth inhibition associated with reduced global DNA methylation, altered promoter methylation of P53, BCL2, and VEGF, and greater DNMT3A/DNMT3B downregulation by (20S)-Rg3; no mechanistic perturbation experiments were reported. | ^24^ | |
| **Rg3** | Hep1-6 implanted in mouse liver | **10 mg/kg by oral feeding, 30 days** | orthotopic tumor growth ↓; survival ↑; tumor shrinkage described | In vivo antitumor activity associated with increased tumor-cell apoptosis and reduced CD105-positive microvessel density, consistent with concomitant pro-apoptotic and anti-angiogenic effects; no pathway-specific mechanistic experiments were reported. | ^25^ | |
| **Rg3** | HepG2 and MHCC-97L cells; subcutaneous xenografts BABL/c nude mice | 0-60 µg/ml for 12–48 h; 2.5-10 mg/kg by oral gavage, 3 weeks | **Cells:** migration ↓; invasion ↓. **Animals:** tumor growth in xenografts ↓ | ARHGAP9 upregulation–associated suppression of migration, invasion, and tumor growth; ARHGAP9 knockdown significantly impaired these Rg3 effects, supporting a functional role for ARHGAP9. | ^26^ | |
| **Rg3** | SMMC-7721 and SK-Hep-1 cells | 8 µg/ml | proliferation ↓; migration ↓; invasion ↓. | HOTAIR downregulation–associated suppression of PI3K/AKT signaling and MMP2/MMP9 expression; HOTAIR overexpression reversed the anti-proliferative, anti-migratory, and anti-invasive effects and restored these molecular changes, supporting a functional role for HOTAIR in Rg3 activity. | ^27^ | |
| **Rg3** | subcapsular parenchyma of the medial liver lobe Hepa1-6 in mice | 10 or 30 mg/kg, intraperitoneal injection, twice a week, 2 weeks; | tumor growth ↓; tumor volume measured and suppressed | In vivo antitumor activity associated with reduced MDSC accumulation and restoration of Kupffer-cell antigen-presenting features (CD86 and MHCII ↑; CD274 ↓); MDSC–Kupffer-cell co-culture findings were consistent with an MDSC-mediated immunosuppressive interaction, but causal in vivo pathway perturbation was not reported. | ^28^ | |

**Note:** HepG2 is generally considered a hepatoblastoma-derived cell line and is sometimes presented separately from established HCC models, although it is frequently used as an HCC-like in vitro model. Accordingly, studies using HepG2 were retained only as supportive liver-tumor–relevant evidence and were not treated as equivalent components of the main HCC evidence base, with limited transferability to established HCC settings. The identity and tumor origin of SK-HEP-1 cells have been debated; therefore, findings from this cell line should be interpreted cautiously. **Abbreviations** HCC = hepatocellular carcinoma; Rg3 = ginsenoside Rg3; 20(S)-Rg3 = 20(S) stereoisomer of Rg3; CTX = CTX (as reported in the source studies); TRAIL = TNF-related apoptosis-inducing ligand; Hep1-6, HepG2, Hep3B, SMMC-7721, Huh-7, H22, MHCC-97L = human or mouse hepatoma cell lines / tumor model cells (as specified); C57BL/6, BABL/c nude mice = mouse strains or nude xenograft hosts (as specified); “in vitro” = cell-based assays.

**Supplementary Table 3. Preclinical studies of compound K (CK) and its derivatives in hepatocellular carcinoma (HCC) models.**

| **Compound** | **Model** | **Dose, time, and route** | **Results** | **Mechanistic implication** | **Refer**  **ence** |
| --- | --- | --- | --- | --- | --- |
| CK | MHCC97-H cell, subcutaneous MHCC97-H xenografts in the left flank of BALB/c (nu/nu) nude mice, with spontaneous liver metastasis | 25–75 μM; 10, 50, or 100 μg/mouse/day by gastric perfusion every other day for 5 weeks | In vitro: colony formation ↓; adhesion ↓; invasion ↓. In vivo: spontaneous metastatic growth inhibited | Anti-metastatic activity associated with time-dependent nuclear export of NF-κB p65 and reduced MMP2/MMP9 expression, consistent with suppression of NF-κB signaling; no pathway-specific perturbation experiments were reported. | ^29^ |
| CK | MHCC97-H | 25–100 µM | Proliferation inhibited; apoptotic sub-G1 fraction increased; mitochondrial membrane potential decreased; DNA damage increased | Apoptosis associated with Fas/FasL upregulation, an increased Bax/Bcl-2 ratio, reduced pro-caspase-9/pro-caspase-3 levels, mitochondrial membrane-potential loss, and reduced Akt phosphorylation, consistent with extrinsic Fas- and intrinsic mitochondrial caspase-dependent apoptotic signaling; no mechanistic perturbation experiments were reported. | ^30^ |
| CK octyl ester | Murine H22 hepatoma cells | 0-100 µM; 10 mg/kg, 14days | In vitro: cytotoxicity increased (dose-dependent). In vivo: H22 tumor cellular apoptosis increased; anti-tumor efficacy with tumor inhibition | Caspase-dependent apoptosis, supported by reversal of M1-O cytotoxicity by the pan-caspase inhibitor Z-VAD-FMK and accompanied by altered Bcl-2/Bax expression and increased cleaved caspase-3. Alleviation of cyclophosphamide-associated spleen and thymus atrophy suggested a potential immunoprotective effect. | ^31^ |
| CK | HepG2 and SMMC-7721 cells; female BALB/c nude mice bearing subcutaneous SMMC-7721 xenografts | 0-60 µM for 48h; 5- 20 mg/kg by intravenously once daily, 15days | In vitro: cell growth ↓; colony formation ↓; apoptosis ↑. In vivo: xenograft growth inhibited (little toxicity reported) | STAT3 inhibition–associated activation of all three unfolded-protein-response branches, endoplasmic-reticulum stress, and apoptosis; CK reduced STAT3 phosphorylation and DNA-binding activity, while CRISPR/Cas9-mediated STAT3 silencing enhanced CK-induced ER stress and apoptosis, supporting a functional role for STAT3 suppression. | ^32^ |
| CK | HepG2 | 6 µM | Cell viability ↓; plate clone formation ↓; cytotoxicity/apoptosis increased | Direct Annexin A2 target engagement, supported by molecular docking and thermal-shift assays; CK disrupted the Annexin A2–NF-κB p50 interaction and nuclear colocalization, thereby suppressing NF-κB activation and downstream gene expression and activating caspase-9/-3. The CK-binding-deficient Annexin A2-K320A mutant conferred resistance to CK, supporting a functional role for Annexin A2 targeting. | ^33^ |
| CK | Bel-7404 and Huh7 cells; rat primary liver cancer model induced by diethylnitrosamine | 20-80 µM for 24-72h; 2.5 or 5 mg/kg intraperitoneally for 2 weeks | In vitro: proliferation ↓; colony formation ↓; G0/G1 arrest; apoptosis increased (apoptosis inferred as part of inhibited growth). In vivo: tumor tissue volumes reduced (tumor volume ↓) | Bclaf1 downregulation–associated HIF-1α ubiquitination/degradation and suppression of HIF-1α-mediated glycolysis; Bclaf1 knockout enhanced these effects, supporting a functional role for Bclaf1. | ^34^ |
| CK | HepG2 and Huh7 cells | 0-10 µM | Sub-G1 increased; apoptosis-related phenotype increased; overall cytotoxicity/apoptosis ↑ | Apoptosis associated with suppression of AKT/mTOR/c-Myc signaling and glycolytic enzymes HK2/PKM2; modulation by LY294002 and pyruvate supported involvement of the AKT/mTOR/c-Myc–glycolysis axis. | ^35^ |
| CK | HepG2 and SK-Hep-1 cells; HepG2 cell-transplanted tumor model in female BALB/c nude mice | 20-60 µM for 48 h; 5, 10, or 20 mg/kg was administered once daily; tumor size was measured every 3 days until tumor volume reached 1,500 mm^3^ | In vitro: proliferation inhibited; ferroptosis induced (cell death phenotype). In vivo: transplanted tumor growth inhibited | Ferroptosis associated with decreased FOXO1 phosphorylation and downregulation of SLC7A11 and GPX4; Ferrostatin-1 and the FOXO1 inhibitor AS1842856 supported involvement of the FOXO1/ferroptosis axis. | ^36^ |
| CK | HepG2 and Hep3B cells; x subcutaneous HepG2 xenografts in nude mice | 5-20 µM for 24 h; 2 or 4 mg/kg by oral gavage daily for 30 days; | In vitro: proliferation inhibited; ferroptosis induced (cell death phenotype). In vivo: xenograft tumor growth reduced | OTUB2 inhibition–mediated ubiquitin-dependent GPX4 degradation, leading to ferroptosis; rescue by NAC/Trolox or GPX4 overexpression and genetic OTUB2 manipulation supported the functional OTUB2/GPX4 ferroptosis axis. | ^37^ |
| CK | HepG2, Huh-7, SMMC-7721, HCC-LM3, and HLE HCC cells; male BALB/c nude mice bearing subcutaneous HCC-LM3 xenografts | 2.5–100 μM; 30 or 60 mg/kg by oral gavage daily for 6 weeks | Under hypoxia: proliferation ↓, colony formation ↓, metastatic potential ↓; EMT-related changes ↓ (EMT marker expression/cytokine-related phenotype ↓). In vivo: xenograft tumor growth ↓; EMT-associated marker expression ↓ | Suppression of the HIF-1α/NF-κB feedback pathway, accompanied by reduced IκB phosphorylation and decreased EMT-related protein and cytokine expression; no pathway-specific perturbation experiments were reported. | ^38^ |
| CK | HepG2, HCC-LM3, and HLE cells; male BALB/c nude mice bearing subcutaneous HCC-LM3 xenografts | 5–100 μM; 90 mg/kg intragastrically for 15 days | Compared with CK: proliferation inhibition at lower concentration; cell cycle arrest **G2/M phase**; apoptosis increased (AO/EB, TUNEL, flow cytometry phenotypes). In vivo: tumor growth ↓; no obvious side effects on main organs | Hippo pathway modulation, accompanied by increased YAP1 phosphorylation and reduced TEAD2 expression in HCC cells and xenograft tissues; transcriptomic and protein-expression findings were consistent with inhibition of YAP1/TEAD2 transcriptional activity, although direct pathway perturbation experiments were not reported. | ^39^ |
| CK | **MHCC97 and Hep3B cells**; **MHCC97 xenografts in male NOD/SCID mice** | 10 μM for 24-72 h; 50 mg/kg every other day for 4 weeks | In vitro: proliferation/colony formation ↓; migration and invasion ↓; apoptosis increased; cell cycle distribution altered. In vivo: tumor formation/volume ↓; **Ki67 ↓**; NK cells & dendritic cells proportions in tumor tissues changed | EZH2 downregulation–associated activation of the dendritic cell–NK cell axis and tumor-immune-microenvironment remodeling; EZH2 overexpression reversed CK-mediated inhibition of HCC-cell proliferation, migration, and invasion, supporting a functional role for EZH2 suppression. | ^40^ |

**Note:** HepG2 is generally considered a hepatoblastoma-derived cell line and is sometimes presented separately from established HCC models, although it is frequently used as an HCC-like in vitro model. Accordingly, studies using HepG2 were retained only as supportive liver-tumor–relevant evidence and were not treated as equivalent components of the main HCC evidence base, with limited transferability to established HCC settings. The identity and tumor origin of SK-HEP-1 cells have been debated; therefore, findings from this cell line should be interpreted cautiously. **Abbreviations** HCC = hepatocellular carcinoma; CK = compound CK; HepG2 = human hepatocellular carcinoma cell line; SMMC-7721, MHCC97-H, Bel-7404, Huh7 = human hepatoma cell lines; H22, M1-O = murine hepatoma/tumor model cells; EMT = epithelial–mesenchymal transition; G0/G1 = cell cycle phases; CoCl2 = cobalt(II) chloride used to induce chemical hypoxia; TNFα = tumor necrosis factor alpha; NK cells/DC = natural killer cells/dendritic cells; TUNEL, AO/EB = apoptosis-related assays/readouts as reported; ferroptosis = iron-dependent regulated cell death; “hypoxia” = hypoxia conditions as implemented in the included studies.

**Supplementary Table 4. Preclinical studies of ginsenoside Rk1 in hepatocellular carcinoma (HCC) models.**

| **Ginsenoside** | **Model** | **Dose, time, and route** | **Results** | **Mechanistic implication** | **Reference** |
| --- | --- | --- | --- | --- | --- |
| **Rk1** | **HepG2 cells** | 12.5-100 µM | Cell growth inhibited; apoptosis induced (caspase-8/-3 activation reported); morphological changes observed; telomerase activity inhibited | Growth inhibition associated with reduced hTERT and c-Myc mRNA expression, unchanged hTR expression, and caspase-8/-3 activation; ERK activation was increased, whereas FADD expression was decreased. These findings are consistent with telomerase inhibition and caspase-dependent apoptosis, although causal mechanistic perturbation experiments were not reported. | ^41^ |
| **Rk1** | **HepG2 cells** | **0-100 µM** | Early stage: **G (1) arrest** and **autophagy**; apoptosis not induced at early stage; **Rk1 + autophagy inhibitor (bafilomycin A1 or beclin 1 siRNA)** enhances anti-tumor effect / apoptosis | Cytoprotective autophagy induced by Rk1, accompanied by LC3-I-to-LC3-II conversion and autolysosome formation; pharmacological autophagy inhibition or beclin 1 silencing enhanced Rk1-induced apoptosis, supporting a functional survival role for autophagy. | ^42^ |
| **Rk1** | **MHCC-97H** | 0–50 µg/mL | Cell viability inhibited; apoptosis increased; mitochondrial depolarization and apoptotic morphology changes described | Intrinsic mitochondrial apoptosis, accompanied by Bax/Bak translocation, cytochrome c and Smac release, reduced anti-apoptotic protein levels, sequential caspase-9/-3 activation, and PARP cleavage. Network analyses implicated MAPK and NF-κB pathways, but no pathway-specific perturbation experiments were reported. | ^43^ |
| **Rk1** | HepG2 and LM3 cells; LM3 subcutaneous xenografts in male nude mice | 50-125 µM for 24-48h, 25 or 50 mg/kg/d (i.p.) for 4 weeks | Proliferation inhibited; **cell cycle arrest and apoptosis** induced in vitro; in vivo anti-tumor effect | ERK/c-Myc pathway suppression–associated downregulation of GLS1 and inhibition of glutamine metabolism, leading to reduced GSH production, ROS accumulation, and apoptosis; transcriptomic and expression findings supported this mechanism, but pathway-specific perturbation experiments were not reported. | ^44^ |
| **Rk1** | **HepG2 cells** | **10-150uM** | Ferroptosis induced (cell viability decreased; colony formation reduced) | FSP1-dependent ferroptosis, accompanied by GSH depletion and increased MDA and intracellular iron levels; rescue by ferroptosis inhibitors (Fer-1 and Lip-1) supported ferroptotic cell death. FSP1 overexpression or silencing modulated Rk1-induced ferroptosis, supporting a functional role for FSP1. | ^45^ |
| **Rk1** | HepG2 cells; primary HCC induced in 2-week-old male C57BL/6 mice by DEN plus CCl₄ | 50-100µM for 24h ;30/60 mg/kg (i.g.), 8 weeks | HCC development/progression inhibited; liver injury/fibrosis/cirrhosis reduced | AMPK/mTOR pathway activation–associated toxic autophagy and apoptosis, accompanied by AMPK activation, LC3-II upregulation, Bcl-2 downregulation, and caspase-cascade activation. Autophagy inhibitors attenuated Rk1-induced apoptosis, supporting a functional role for autophagy-dependent apoptosis. | ^46^ |

**Note:** HepG2 is generally considered a hepatoblastoma-derived cell line and is sometimes presented separately from established HCC models, although it is frequently used as an HCC-like in vitro model. Accordingly, studies using HepG2 were retained only as supportive liver-tumor–relevant evidence and were not treated as equivalent components of the main HCC evidence base, with limited transferability to established HCC settings. The identity and tumor origin of SK-HEP-1 cells have been debated; therefore, findings from this cell line should be interpreted cautiously. **Abbreviations** HCC = hepatocellular carcinoma; Rk1 = ginsenoside Rk1; HepG2 = human hepatocellular carcinoma cell line; MHCC-97H = highly metastatic human HCC cell line; DEN = diethylnitrosamine used to induce liver cancer in animals; CCl4 = carbon tetrachloride used to induce liver injury/fibrosis in animals; G(1) = G1 phase of cell cycle; autophagy = autophagy-related phenotype as assessed in included studies; apoptosis = apoptosis readouts as reported; bafilomycin A1 = autophagy inhibitor (when mentioned); ferroptosis = iron-dependent regulated cell death; EMT = epithelial–mesenchymal transition; DAPI = apoptosis-related fluorescence staining assay/readout; MTT = cell viability assay; IC50 = half-maximal inhibitory concentration; caspase-8/-3 = apoptosis-related caspase activation markers; telomerase activity = telomerase activity marker as reported.

**Supplementary table 5 Preclinical studies of additional ginsenosides and derivatives in hepatocellular carcinoma (HCC) models.**

| **Ginsenoside** | **Model** | **Dose, time, and route** | **Results** | **Mechanistic implication** | **Reference** |
| --- | --- | --- | --- | --- | --- |
| Rg1 | **SK-HEP-1** | 0-100 µM | Cell proliferation/DNA synthesis ↑; cyclin E/cdk2-related kinase activity ↑ (stimulated cell growth) | Growth-promoting activity associated with increased cyclin E and CDK2 protein expression and enhanced cyclin E-dependent kinase activity. Cycloheximide blocked Rg1-stimulated DNA synthesis, supporting a requirement for de novo protein synthesis; no pathway-specific genetic perturbation experiments were reported. | ^47^ |
| Rg1 | **HepG2** | 50-400 µM | TGF-β1–induced invasion/migration ↓; EMT initiation suppressed; E-cadherin ↑, vimentin ↓ | Anti-invasive and anti-migratory effects associated with inhibition of TGF-β1-induced EMT; no pathway-specific mechanistic perturbation experiments were reported. | ^48^ |
| Rg1 | Huh7 and MHCC97H cells; subcutaneous injection of HCC  cells in BALB/c nude mice | 0-100 µmol/L; 50 mg/kg via intraperitoneal  injection, once a day, three days | Proliferation ↓, migration capability ↓; ferroptosis phenotype induced; SLC7A11 expression ↓ | Ferroptosis induction associated with SLC7A11 downregulation and restored ferroptosis sensitivity. SLC7A11 was proposed as a downstream mediator, but its functional role requires further validation; no mechanistic perturbation experiments were reported. | ^49^ |
| Rg5 | **HepG2** | 10 mg/mL | PRDX1 inhibition → ROS ↑; mitochondrial ROS accumulation ↑; mitochondrial/ intrinsic-apoptosis phenotype; cell survival ↓ | Direct PRDX1 targeting, with Rg5 binding to the PRDX1 Asn145 residue and suppressing its peroxidase activity, disrupted redox homeostasis, resulting in mitochondrial ROS accumulation and intrinsic apoptosis. Rg5 also showed synergistic antitumor activity with doxorubicin. | ^50^ |
| Rs4 | **SK-HEP-1** | 0-25 µM | Cell cycle arrest at G1/S followed by apoptosis ↑; apoptosis peak, DNA fragmentation | Apoptosis and G1/S arrest were associated with increased p53 and p21^WAF1^ protein levels and reduced cyclin E- and cyclin A-dependent kinase activities; no pathway-specific mechanistic perturbation experiments were reported. | ^51^ |
| F2 | HepG2; subcutaneously  injected H22 cells in male ICR mice | 0-120 μM; 100 mg/kg/day via intragastric gavage, 2 weeks | HCC growth inhibition: in vitro proliferation/growth ↓; in vivo tumor growth inhibited; STAT3 activity ↓ | Direct STAT3 targeting was supported by molecular docking, SPR, and NMR. F2 reduced STAT3 Y705 phosphorylation, nuclear translocation, and transcriptional activity, supporting STAT3 inhibition as a functional mechanism underlying its anti-HCC activity. | ^52^ |
| Rb | **HepG2 and murine H22 cells**; lung metastasis mouse model | 100–250 µg/mL; 7 mg/kg or 35 mg/kg, 3weeks | Migration/invasion ↓; EMT-related markers (N-cadherin/Snail/MMP9) ↓; lung metastasis nodules ↓ | Anti-metastatic activity associated with disruption of platelet–tumor cell interactions and attenuation of platelet activation, accompanied by suppression of platelet-associated EMT features. No specific mechanistic perturbation experiments were reported. | ^53^ |
| Rh4 | HUH7 and LM3 cells; HCC xenograft tumors in male BALB/c nude mice | 40 -80 µM for 30 min; | Under LPS conditions: cell viability ↓; Ki67 ↓; migration/invasion ↓; in vivo tumor growth suppressed | Anti-inflammatory HCC effects were associated with downregulation of HDAC4, IL-6, and STAT3 phosphorylation, accompanied by suppression of glycolytic features. HDAC4 overexpression alleviated Rh4-mediated inhibition of proliferation, migration, invasion, and glycolysis, supporting a functional role for the HDAC4/IL-6/STAT3 axis. | ^54^ |
| Rk3 | **HepG2 and HCC-LM3 cells**; primary liver cancer mice + subcutaneous tumor model | Rk3 for 24 h; 50-100 mg/kg via i.p., 28 days | Proliferation ↓; G1-phase arrest; autophagy and apoptosis phenotypes; in vivo tumor growth inhibition | PI3K/AKT targeting was supported by molecular docking and SPR, with proteomic and siRNA evidence supporting a functional role for PI3K/AKT pathway regulation in Rk3-mediated HCC growth inhibition, autophagy, and apoptosis. | ^55^ |
| (20S)-APPT | Hep3B, SNU387, and SK-HEP-1 cells; SK-HEP-1 and Hep3B xenograft models in female immunodeficient mice | 10 μM; 200 mg/kg p.o. daily for 24–27 days | Ferroptosis induced; in vivo tumor growth suppressed | Direct FSP1 targeting/inhibition promoted ACSL4-dependent arachidonic-acid oxidation and mitochondrial ROS production, leading to ferroptosis. Combined FSP1 and GCS inhibition synergistically induced ferroptosis in otherwise resistant cancer cells while sparing noncancerous cells. | ^56^ |
| Protopanaxadiol (PPD) | HepG2; PLC/PRF/5 xenograft with lung metastasis in Female BALB/C nude mice | 20-40 µM; 30 mg/kg via intragastrically administered every day, 6 weeks | Proliferation ↓; migration and invasion ↓; in vivo tumor volume ↓ and lung metastasis ↓ | Direct STAT3 targeting was supported by molecular docking and Biacore binding assays. PPD reduced STAT3 phosphorylation and nuclear translocation, accompanied by Twist1 downregulation and EMT suppression, consistent with inhibition of the STAT3/Twist1 axis; no STAT3-specific genetic perturbation experiments were reported. | ^57^ |
| Rd | **HepG2** | 0-200 µM | Migration/invasion ↓; MMP activation/expression ↓; in vivo metastasis suppressed | Anti-migratory and anti-invasive effects were associated with MAPK/AP-1 inactivation, reduced MMP expression/activity, and enhanced focal-adhesion formation. | ^58^ |
| (20S)-Protopanaxadiol (PPD) | **HepG2** | 0 -80 µM | Cell growth inhibited and apoptosis induced; clear ER-stress–associated apoptosis phenotype | ER-stress/UPR activation accompanied intrinsic and extrinsic apoptotic signaling. CHOP knockdown significantly attenuated apoptosis and reversed PPD-associated changes in Bcl-2, DR5, caspase-8, and PARP, supporting a functional role for CHOP-mediated ER-stress signaling; knockdown of individual UPR branches did not affect apoptosis. | ^59^ |

**Note:** HepG2 is generally considered a hepatoblastoma-derived cell line and is sometimes presented separately from established HCC models, although it is frequently used as an HCC-like in vitro model. Accordingly, studies using HepG2 were retained only as supportive liver-tumor–relevant evidence and were not treated as equivalent components of the main HCC evidence base, with limited transferability to established HCC settings. The identity and tumor origin of SK-HEP-1 cells have been debated; therefore, findings from this cell line should be interpreted cautiously. **Abbreviations** HCC = hepatocellular carcinoma; EMT = epithelial–mesenchymal transition; G1/S = cell-cycle phase transition; Ki-67 = proliferation marker; ROS = reactive oxygen species; STAT3 = signal transducer and activator of transcription 3; TGF-β1 = transforming growth factor-β1; LPS = lipopolysaccharide; HB = hepatoblastoma; ferroptosis = iron-dependent regulated cell death; cyclin E/cdk2-related kinase activity = cyclin E–CDK2 axis; PRDX1 = peroxiredoxin 1; Akt = protein kinase B; CYP1A1 = cytochrome P450 1A1; AhR = aryl hydrocarbon receptor; XRE = xenobiotic-responsive element; E-cadherin = epithelial marker; vimentin = mesenchymal marker; SLC7A11 = solute carrier family 7 member 11; N-cadherin, Snail, MMP9 = EMT/metastasis-related markers; (20S)-APPT and (20S)-Protopanaxadiol = stereoisomer nomenclature as used in the original papers.

1	Park, J. A., Lee, K. Y., Oh, Y. J., Kim, K. W. & Lee, S. K. Activation of caspase-3 protease via a Bcl-2-insensitive pathway during the process of ginsenoside Rh2-induced apoptosis. *Cancer Lett* **121**, 73–81 (1997). <https://doi.org/10.1016/s0304-3835(97)00333-9>

2	Guo, X. X. *et al.* Ginsenoside Rh2 induces human hepatoma cell apoptosisvia bax/bak triggered cytochrome C release and caspase-9/caspase-8 activation. *Int J Mol Sci* **13**, 15523–15535 (2012). <https://doi.org/10.3390/ijms131215523>

3	Park, H. M., Kim, S. J., Kim, J. S. & Kang, H. S. Reactive oxygen species mediated ginsenoside Rg3- and Rh2-induced apoptosis in hepatoma cells through mitochondrial signaling pathways. *Food Chem Toxicol* **50**, 2736–2741 (2012). <https://doi.org/10.1016/j.fct.2012.05.027>

4	Shi, Q. *et al.* Effect of ginsenoside Rh2 on the migratory ability of HepG2 liver carcinoma cells: recruiting histone deacetylase and inhibiting activator protein 1 transcription factors. *Mol Med Rep* **10**, 1779–1785 (2014). <https://doi.org/10.3892/mmr.2014.2392>

5	Chen, W. & Qiu, Y. Ginsenoside Rh2 Targets EGFR by Up-Regulation of miR-491 to Enhance Anti-tumor Activity in Hepatitis B Virus-Related Hepatocellular Carcinoma. *Cell Biochem Biophys* **72**, 325–331 (2015). <https://doi.org/10.1007/s12013-014-0456-9>

6	Lv, Q. *et al.* Antitumoral Activity of (20R)- and (20S)-Ginsenoside Rh2 on Transplanted Hepatocellular Carcinoma in Mice. *Planta Med* **82**, 705–711 (2016). <https://doi.org/10.1055/s-0042-101764>

7	Shi, Q. *et al.* Anticancer effect of 20(S)-ginsenoside Rh2 on HepG2 liver carcinoma cells: Activating GSK-3β and degrading β-catenin. *Oncol Rep* **36**, 2059–2070 (2016). <https://doi.org/10.3892/or.2016.5033>

8	Yang, Z., Zhao, T., Liu, H. & Zhang, L. Ginsenoside Rh2 inhibits hepatocellular carcinoma through β-catenin and autophagy. *Sci Rep* **6**, 19383 (2016). <https://doi.org/10.1038/srep19383>

9	Li, Q., Li, B., Dong, C., Wang, Y. & Li, Q. 20(S)-Ginsenoside Rh2 suppresses proliferation and migration of hepatocellular carcinoma cells by targeting EZH2 to regulate CDKN2A-2B gene cluster transcription. *Eur J Pharmacol* **815**, 173–180 (2017). <https://doi.org/10.1016/j.ejphar.2017.09.023>

10	Chen, W., Chu, S., Li, H. & Qiu, Y. MicroRNA-146a-5p enhances ginsenoside Rh2-induced anti-proliferation and the apoptosis of the human liver cancer cell line HepG2. *Oncol Lett* **16**, 5367–5374 (2018). <https://doi.org/10.3892/ol.2018.9235>

11	Chen, W. W. *et al.* Microarray analysis of altered long non-coding RNA expression profile in liver cancer cells treated by ginsenoside Rh2. *J Asian Nat Prod Res* **21**, 742–753 (2019). <https://doi.org/10.1080/10286020.2018.1490273>

12	Zhang, J. *et al.* Transcriptome Analyses of the Anti-Proliferative Effects of 20(S)-Ginsenoside Rh2 on HepG2 Cells. *Front Pharmacol* **10**, 1331 (2019). <https://doi.org/10.3389/fphar.2019.01331>

13	Chen, C. *et al.* (20S) Ginsenoside Rh2 Exerts Its Anti-Tumor Effect by Disrupting the HSP90A-Cdc37 System in Human Liver Cancer Cells. *Int J Mol Sci* **22** (2021). <https://doi.org/10.3390/ijms222313170>

14	Qiang, S. Q. *et al.* Anticancer Effect of Rh2, a Histone Deacetylase Inhibitor, in HepG2 Cells and HepG2 Cell-Derived Xenograft Tumors Occurs via the Inhibition of HDACs and Activation of the MAPK Signaling Pathway. *Asian Pac J Cancer Prev* **22**, 2529–2539 (2021). <https://doi.org/10.31557/apjcp.2021.22.8.2529>

15	Wang, Y. S., Chen, C., Zhang, S. Y., Li, Y. & Jin, Y. H. (20S) Ginsenoside Rh2 Inhibits STAT3/VEGF Signaling by Targeting Annexin A2. *Int J Mol Sci* **22** (2021). <https://doi.org/10.3390/ijms22179289>

16	Liu, W. *et al.* 20(s)-ginsenoside Rh2 promotes TRAIL-induced apoptosis by upregulating DR5 in human hepatocellular carcinoma cells. *Med Oncol* **39**, 70 (2022). <https://doi.org/10.1007/s12032-022-01663-6>

17	Hu, Q. R. *et al.* Ginsenoside Rh2 and its octyl ester derivative inhibited invasion and metastasis of hepatocellular carcinoma via the c-Jun/COX2/PGE2 pathway. *Phytomedicine* **121**, 155131 (2023). <https://doi.org/10.1016/j.phymed.2023.155131>

18	Xu, L. *et al.* 20(S)-Ginsenoside Rh2 inhibits hepatocellular carcinoma by suppressing angiogenesis and the GPC3-mediated Wnt/β‑catenin signaling pathway. *Acta Biochim Biophys Sin (Shanghai)* **56**, 688–696 (2024). <https://doi.org/10.3724/abbs.2024038>

19	Park, J., Jeong, I., Han, A. & Kim, O. K. Ginsenoside Rh2 Enhances CD8(+) T Cell-Mediated Anticancer Immunity in Hepatocellular Carcinoma. *Nutrients* **18** (2026). <https://doi.org/10.3390/nu18142224>

20	Jiang, J. W., Chen, X. M., Chen, X. H. & Zheng, S. S. Ginsenoside Rg3 inhibit hepatocellular carcinoma growth via intrinsic apoptotic pathway. *World J Gastroenterol* **17**, 3605–3613 (2011). <https://doi.org/10.3748/wjg.v17.i31.3605>

21	Zhang, C. *et al.* Antitumor effects of ginsenoside Rg3 on human hepatocellular carcinoma cells. *Mol Med Rep* **5**, 1295–1298 (2012). <https://doi.org/10.3892/mmr.2012.808>

22	Lee, J. Y. *et al.* Sensitization of TRAIL-induced cell death by 20(S)-ginsenoside Rg3 via CHOP-mediated DR5 upregulation in human hepatocellular carcinoma cells. *Mol Cancer Ther* **12**, 274–285 (2013). <https://doi.org/10.1158/1535-7163.Mct-12-0054>

23	Wu, R., Ru, Q., Chen, L., Ma, B. & Li, C. Stereospecificity of ginsenoside Rg3 in the promotion of cellular immunity in hepatoma H22-bearing mice. *J Food Sci* **79**, H1430–1435 (2014). <https://doi.org/10.1111/1750-3841.12518>

24	Teng, S. *et al.* Effects of R type and S type ginsenoside Rg3 on DNA methylation in human hepatocarcinoma cells. *Mol Med Rep* **15**, 2029–2038 (2017). <https://doi.org/10.3892/mmr.2017.6255>

25	Hu, S. *et al.* Ginsenoside Rg3 Prolongs Survival of the Orthotopic Hepatocellular Carcinoma Model by Inducing Apoptosis and Inhibiting Angiogenesis. *Anal Cell Pathol (Amst)* **2019**, 3815786 (2019). <https://doi.org/10.1155/2019/3815786>

26	Sun, M. Y. *et al.* Ginsenoside Rg3 inhibits the migration and invasion of liver cancer cells by increasing the protein expression of ARHGAP9. *Oncol Lett* **17**, 965–973 (2019). <https://doi.org/10.3892/ol.2018.9701>

27	Pu, Z. *et al.* Ginsenoside-Rg3 inhibits the proliferation and invasion of hepatoma carcinoma cells via regulating long non-coding RNA HOX antisense intergenic. *Bioengineered* **12**, 2398–2409 (2021). <https://doi.org/10.1080/21655979.2021.1932211>

28	Zhao, H., Liu, J., Zhang, X., Xu, J. & Zhai, X. Bioinformatics and experimental verification to explore the potential mechanism of ginsenoside Rg3 suppresses hepatocellular carcinoma progression. *Int Immunopharmacol* **143**, 113543 (2024). <https://doi.org/10.1016/j.intimp.2024.113543>

29	Ming, Y. *et al.* Ginsenoside compound K attenuates metastatic growth of hepatocellular carcinoma, which is associated with the translocation of nuclear factor-κB p65 and reduction of matrix metalloproteinase-2/9. *Planta Med* **77**, 428–433 (2011). <https://doi.org/10.1055/s-0030-1250454>

30	Zheng, Z. Z. *et al.* Compound K-induced apoptosis of human hepatocellular carcinoma MHCC97-H cells in vitro. *Oncol Rep* **32**, 325–331 (2014). <https://doi.org/10.3892/or.2014.3171>

31	Hou, J. *et al.* Octyl ester of ginsenoside compound K as novel anti-hepatoma compound: Synthesis and evaluation on murine H22 cells in vitro and in vivo. *Chem Biol Drug Des* **91**, 951–956 (2018). <https://doi.org/10.1111/cbdd.13153>

32	Zhang, X. *et al.* Compound K Induces Endoplasmic Reticulum Stress and Apoptosis in Human Liver Cancer Cells by Regulating STAT3. *Molecules* **23** (2018). <https://doi.org/10.3390/molecules23061482>

33	Wang, Y. S. *et al.* Ginsenoside compound K inhibits nuclear factor-kappa B by targeting Annexin A2. *J Ginseng Res* **43**, 452–459 (2019). <https://doi.org/10.1016/j.jgr.2018.04.002>

34	Zhang, S. *et al.* Ginsenoside Compound K Regulates HIF-1α-Mediated Glycolysis Through Bclaf1 to Inhibit the Proliferation of Human Liver Cancer Cells. *Front Pharmacol* **11**, 583334 (2020). <https://doi.org/10.3389/fphar.2020.583334>

35	Shin, N. *et al.* Apoptotic effect of compound K in hepatocellular carcinoma cells via inhibition of glycolysis and Akt/mTOR/c-Myc signaling. *Phytother Res* **35**, 3812–3820 (2021). <https://doi.org/10.1002/ptr.7087>

36	Chen, J. *et al.* Ginsenoside compound K induces ferroptosis via the FOXO pathway in liver cancer cells. *BMC Complement Med Ther* **24**, 174 (2024). <https://doi.org/10.1186/s12906-024-04471-9>

37	Jiang, Y. *et al.* The Achilles' heel of hepatocellular carcinoma: ginsenoside compound K as a novel GPX4 degrader promotes ferroptosis in hepatocellular carcinoma. *J Transl Med* **24**, 277 (2026). <https://doi.org/10.1186/s12967-025-07587-9>

38	Zhang, J., Ma, X. & Fan, D. Ginsenoside CK Inhibits Hypoxia-Induced Epithelial-Mesenchymal Transformation through the HIF-1α/NF-κB Feedback Pathway in Hepatocellular Carcinoma. *Foods* **10** (2021). <https://doi.org/10.3390/foods10061195>

39	Zhang, J. *et al.* A derivant of ginsenoside CK and its inhibitory effect on hepatocellular carcinoma. *Life Sci* **304**, 120698 (2022). <https://doi.org/10.1016/j.lfs.2022.120698>

40	Ye, F. *et al.* Network Pharmacology and Transcriptomic Analysis Reveal the Mechanism by Which Ginsenoside CK Remodels the Tumor Immune Microenvironment to Inhibit Liver Cancer Progression. *Chem Biol Drug Des* **106**, e70204 (2025). <https://doi.org/10.1111/cbdd.70204>

41	Kim, Y. J. *et al.* Anti-tumor activity of the ginsenoside Rk1 in human hepatocellular carcinoma cells through inhibition of telomerase activity and induction of apoptosis. *Biol Pharm Bull* **31**, 826–830 (2008). <https://doi.org/10.1248/bpb.31.826>

42	Ko, H., Kim, Y. J., Park, J. S., Park, J. H. & Yang, H. O. Autophagy inhibition enhances apoptosis induced by ginsenoside Rk1 in hepatocellular carcinoma cells. *Biosci Biotechnol Biochem* **73**, 2183–2189 (2009). <https://doi.org/10.1271/bbb.90250>

43	Chen, C., Lv, Q., Li, Y. & Jin, Y. H. The Anti-Tumor Effect and Underlying Apoptotic Mechanism of Ginsenoside Rk1 and Rg5 in Human Liver Cancer Cells. *Molecules* **26** (2021). <https://doi.org/10.3390/molecules26133926>

44	Lu, H. *et al.* Ginsenoside Rk1 regulates glutamine metabolism in hepatocellular carcinoma through inhibition of the ERK/c-Myc pathway. *Food Funct* **13**, 3793–3811 (2022). <https://doi.org/10.1039/d1fo03728e>

45	Jiang, Y., Yu, Y., Pan, Z., Wang, Z. & Sun, M. Ginsenoside RK1 Induces Ferroptosis in Hepatocellular Carcinoma Cells through an FSP1-Dependent Pathway. *Pharmaceuticals (Basel)* **17** (2024). <https://doi.org/10.3390/ph17070871>

46	Wu, H. *et al.* Ginsenoside Rk1 induces autophagy-dependent apoptosis in hepatocellular carcinoma by AMPK/mTOR signaling pathway. *Food Chem Toxicol* **186**, 114587 (2024). <https://doi.org/10.1016/j.fct.2024.114587>

47	Lee, K. Y. & Lee, S. K. Ginsenoside-Rg1 positively regulates cyclin E-dependent kinase activity in human hepatoma SK-HEP-1 cells. *Biochem Mol Biol Int* **39**, 539–546 (1996). <https://doi.org/10.1080/15216549600201591>

48	Yu, M. *et al.* Ginsenoside Rg1 attenuates invasion and migration by inhibiting transforming growth factor-β1-induced epithelial to mesenchymal transition in HepG2 cells. *Mol Med Rep* **11**, 3167–3173 (2015). <https://doi.org/10.3892/mmr.2014.3098>

49	Wei, A., Liu, Z., Zhao, F., Zhao, Y. & Liu, B. Ginsenoside Rg1 triggers ferroptosis to inhibit hepatocellular carcinoma via SLC7A11 downregulation. *Discov Oncol* (2026). <https://doi.org/10.1007/s12672-026-05223-6>

50	Ye, H. L. *et al.* Ginsenoside Rg5 Targets PRDX1 to Disrupt Redox Homeostasis and Induce Mitochondria-Dependent Apoptosis in Human Hepatocellular Carcinoma HepG2 Cells. *Molecules* **31** (2026). <https://doi.org/10.3390/molecules31030557>

51	Kim, S. E., Lee, Y. H., Park, J. H. & Lee, S. K. Ginsenoside-Rs4, a new type of ginseng saponin concurrently induces apoptosis and selectively elevates protein levels of p53 and p21WAF1 in human hepatoma SK-HEP-1 cells. *Eur J Cancer* **35**, 507–511 (1999). <https://doi.org/10.1016/s0959-8049(98)00415-8>

52	Tan, X. *et al.* A large-scale transcriptional analysis reveals herb-derived ginsenoside F2 suppressing hepatocellular carcinoma via inhibiting STAT3. *Phytomedicine* **120**, 155031 (2023). <https://doi.org/10.1016/j.phymed.2023.155031>

53	Miao, L., Yang, Y., Cheng, M., Chen, L. & Han, C. Ginsenoside Rb prevents the metastasis of hepatocarcinoma by blocking the platelet-tumor cell interaction. *Naunyn Schmiedebergs Arch Pharmacol* **398**, 1721–1733 (2025). <https://doi.org/10.1007/s00210-024-03387-y>

54	Jiang, R. *et al.* Ginsenoside Rh4 inhibits inflammation-related hepatocellular carcinoma progression by targeting HDAC4/IL-6/STAT3 signaling. *Mol Genet Genomics* **298**, 1479–1492 (2023). <https://doi.org/10.1007/s00438-023-02070-w>

55	Qu, L., Liu, Y., Deng, J., Ma, X. & Fan, D. Ginsenoside Rk3 is a novel PI3K/AKT-targeting therapeutics agent that regulates autophagy and apoptosis in hepatocellular carcinoma. *J Pharm Anal* **13**, 463–482 (2023). <https://doi.org/10.1016/j.jpha.2023.03.006>

56	Liu, F. Y. *et al.* Ginsenoside (20)S-APPT induces ferroptosis in hepatocellular carcinoma and cholangiocarcinoma by targeting FSP1. *Acta Pharmacol Sin* **46**, 3273–3290 (2025). <https://doi.org/10.1038/s41401-025-01589-5>

57	Yang, L. *et al.* Protopanaxadiol inhibits epithelial-mesenchymal transition of hepatocellular carcinoma by targeting STAT3 pathway. *Cell Death Dis* **10**, 630 (2019). <https://doi.org/10.1038/s41419-019-1733-8>

58	Yoon, J. H., Choi, Y. J., Cha, S. W. & Lee, S. G. Anti-metastatic effects of ginsenoside Rd via inactivation of MAPK signaling and induction of focal adhesion formation. *Phytomedicine* **19**, 284–292 (2012). <https://doi.org/10.1016/j.phymed.2011.08.069>

59	Zhu, G. Y. *et al.* 20(S)-Protopanaxadiol, a metabolite of ginsenosides, induced cell apoptosis through endoplasmic reticulum stress in human hepatocarcinoma HepG2 cells. *Eur J Pharmacol* **668**, 88–98 (2011). <https://doi.org/10.1016/j.ejphar.2011.06.008>
